# Supplementary material for: Effectiveness of education intervention, with regards to physical activity level and a healthy diet, among Middle Eastern adolescents in Malaysia: A study protocol for a randomized control trial, based on a health belief model
Source: PLoS One. 2024 Jan 17;19(1):e0289937. doi: 10.1371/journal.pone.0289937 (PMC10793934; doi:10.1371/journal.pone.0289937)
Supplement: S1 File — (PDF) [file pone.0289937.s002.pdf]

FORM 1: APPLICATION FORM

PART A: Brief Details of Project

1 . Research Proposal THE EFFECTIVENESS OF HEALTH LITERACY INTERVENTION ON PHYSICAL ACTIVITY AND HEALTHY DIET AMONG MIDDLE EASTERN ADOLESCENTS IN MALAYSIA

Project Start 09/2022 To 12 2023

2. Principal Investigator

Name :Nik Daliana Nik Farid
Title : Dr
Position :Associate Professor
Telephone : 0122779250
Email : daliana@ummc.edu.my
Department :Department of Social and Preventive Medicine
Academy/Faculty/Institute/Centre : Faculty Of Medicine

3. Co-Investigator (if any)

Name :Mohamad Shafiq Bin Azanan
Title : Dr
Position : Senior Lecturer
Telephone : Click here to enter text.
Email : mohamadshafiq@um.edu.my
Department :Department of Paediatrics
Academy/Faculty/Institute/Centre : Faculty Of Medicine

4. Student Principal Investigator (PI) only

Name :Hanan Hasan Hussein Al-Haroni
Title : Dr
Position :Doctor of Philosophy (PhD) candidate
Telephone : 0172855427
Email : alharonihanan@gmail.com
Department :Social and Preventive Medicine
Academy/Faculty/Institute/Centre: Faculty of Medicine
Degree/Programme :Doctor of Philosophy (PhD)

5. Research funding/Grant :No Funding

6. Amount of Research Grant ꟷ Click here to enter text.

PART B: Data Collection

7. New data to be collected from human participant. Please tick any that apply.

| | |
|---|---|
| C] | Focus group |
| | Experimental procedures/treatment/intervention |
| | Internet survey |
| | Observation |
| | Personal interviews |
| | Telephone survey |
| | Action research |
| | Questionnaire |
| | Others (please state):Click here to enter text. |

8. Existing records with personal data.

| | |
|---|---|
| | Yes |
| | No |

9. Brief description of study.
   i. Background of study (less than 300words).

In Malaysia, Middle Eastern adolescents are going through changes in living status, yet few studies show that overweight and obesity are prevalent among Arabic secondary school students. There have not been any intervention studies among Middle Eastern adolescents in Malaysia. The current study aims to determine the effects of an intervention program on physical activity and healthy diet behaviour among Middle Eastern adolescent students in Arabic schools in Malaysia. Method: A cluster randomized controlled study will be conducted among 250 Middle Eastern adolescent students in Arabic schools in Malaysia. The intervention and control schools will be selected and allocated randomly. The intervention group will have six weeks of fortnightly six sessions (45 minutes), while the control group will have their regular curriculums and normal physical activity routine. Anthropometric questionnaires include knowledge, attitude and practice of lifestyle, physical activity, adolescent sedentary activity, food assessment, eating attitudes test-26 and health belief model questionnaire. Data

will be collected from intervention and control groups at baseline, post-intervention and one month follow-up after the intervention. Data will be analysed by using the (SPSS) software version 25. Descriptive statistics will be used to distribute and summarize the data. The normality of variables will be tested by The Kolmogorov-Smirnov test and the Skewness & Kurtosis test. P-value < 0.05 will considered as statistically significant. One Way ANOVA will be used to determine whether any significant withingroup differences existed over time for the selected variables if the data is normally distributed, while Friedman's test will be used if the data is not normally distributed. Generalized Estimating Equation (GEE) will be used to test the effect of the intervention program for the selected variables (outcomes) between & within-group at baseline, six weeks, and one month after intervention which is adjusted for clustering.

ii. Rationale of study/problem statement

Overweight and obesity are common among Arab adolescents. In Malaysia, there are more than 25 Arabic schools. Many of these schools are public and operated by the Ministry of Education in their respective countries. Among Arabic secondary school students in Kuala Lumpur and Putrajaya, overweight and obesity are prevalent with limited health literacy(Eltayeb et al., 2016).In Malaysia, Middle Eastern adolescents have more access to energized food, as well as a better transportation network that reduces frequent movement. Additionally, they frequently go to western fast food outlets (Mareh et al., 2020). However, very scant research has looked into the contributing factors to their obesity. It is also an interest of the researcher to study the reasons for physical inactivity and unhealthy food behaviour among this population. To the best of our knowledge, there are no intervention studies to reduce body weight currently implemented for Middle Eastern adolescents in Malaysia. This study is one of the first trial studies among Arabic school students in Malaysia. Thus the information provided can help to create a healthy society by displaying areas where personal and social development can be enhanced through further education and training in positive life skills.

iii. Objective(s) of study.

General Objective

To determine the effects of an intervention program on physical activity and healthy diet behaviour among Middle Eastern adolescent students in Arabic schools in Malaysia.

Specific Objectives

1. To determine the demographic characteristics, physical activity level, nutrition status (BMI and dietary intake) and disordered eating among ME adolescent students in Arabic schools in Malaysia.
2. To determine the baseline knowledge, attitude and practice on body weight status among ME adolescent students in Arabic schools in Malaysia.
3. To compare the change of knowledge, attitude, and practice on body weight status, physical activity level, nutrition status (BMI and dietary intake), and disordered eating between intervention and control groups among Middle Eastern adolescent students in Arabic schools in Malaysia.
4. To determine the effect of the intervention on the behavioural intention of weight management, perceived self-efficacy in exercise, perceived self-efficacy in dieting, cue to action, perceived benefits, perceived barriers, perceived susceptibility and perceived severity.

10. Study participants (new data to be collected from human participants).

i. Study sample. Please specify.

The current study will be conducted in four intermediate Arabic schools in Malaysia which will be allocated randomly. The first two schools will be assigned the intervention school and labelled as the intervention group, and the second two schools will be assigned the control school.

The study population will be the Middle Eastern students in early adolescence, which are 13 and 14 years old in grades seven and eight studying in the selected Arabic schools in Klang Valley Area who fulfil the inclusion criteria from September 2022 to October 2022. The total sample size is 250 participants (125 participants for each group).

ii. How will participants be recruited? Please specify.

After selecting the assigning, 2 schools for intervention and 2 control schools and selecting the classes, and according to the inclusion criteria, all subjects have the same characteristics, so they have an equal chance to participate in this study. Therefore, a probability sampling technique using random selection will be used. To illustrate, in each school, the researcher will randomly pick 63 students 13 years old and 63 students 14 years old from each school selected will be the sample of the study. All selections will be done randomly through Excel software. The researcher will utilise the Physical Education class slot.

iii. Who will perform the data collection?

Data will be collected over six weeks from two Arabic schools in Klang Valley Area by the researcher. Only those available at the time of data collection will be further briefed and assessed on their eligibility according to inclusion and exclusion criteria. An invitation will be sent to the parents of the eligible participants by the school administration to obtain the written consent before starting the study. After the researcher receives the written assent form from students and their parents, the researcher will start to collect data. The participant information sheet in English and Arabic will be provided for the eligible students and their parents. The aims and protocol of this study will be explained to the participants. All participants are volunteers in this study, and they understand that they have the right to withdraw from this research at any time without

giving any reason. They will know that this study is confidential. All information provided concerning their identities will remain private by using identifiers. Participants will be asked about socio-demographic information to answer them, anthropometric measures will measure, and they will ask to answer questionnaires. There is no compensation given to participants and no possible risks or adverse effects in this research for participants.

iiii who will collect the data.

The researcher

iv. Participant inclusion criteria (e.g., residents aged 18 years and above).

- The study population consisted of Arabic students.
- Students aged 13 to 14 years.
- Students who agree to participate in the study.
- Students who at least one of their parents consent to participate in the study.

v. Participant exclusion criteria.

- Non-Arabic Students
- Students with known diseases or conditions or on medical treatment that pose risk from or prohibited them of physical activities such as, but not limited to asthma, diabetes, cancer, cardiovascular diseases, fractures, cirrhosis based on information from the parent in the consent form.
- Students with physical disabilities or injuries restrict physical activity.

vi. Are the participants given any form of payment/incentive to participate?

NO

PART C: Risk and Benefits

11. Possible benefits to participants

There is no direct benefit to participants

The intervention group may benefit. The study will increase their awareness of the dangers of negative practices and its eventual negative impact on their health status. As a result, the pupils will maintain a healthy weight, which will benefit them as adults. Additionally, the results of this study will assess the diet and physical activity level, along with other perceived risk factors to prevent the prevalence of obesity.

12. Risk of harm (new data to be collected from human participants).

| RISK | YES | NO |
|---|---|---|
| Will the study involve intervention, such as action research/treatment of any type? If YES, please give details:<br>The intervention will be in term of education program. This program will include educational booklets and educational classes. It will be carried out in a single-blind trial. However, the researchers know about the details of the intervention, but the students were unaware of the presence of the control group or that they will be reevaluated. The education program will be developed by the researcher after consultation with experts in health promotion with a special interest in changing behaviour related to obesity. The educational program will be designed based on the HBM components to improve knowledge and promote lifestyle adherence in terms of healthy diet behaviour and physical activity by focusing on the attitude and beliefs of students with increasing knowledge and perceived risk of diseases.<br>____________________________________ | | |
| Is it possible that the duration of the procedures will cause minimal stress, in particular, for children, given their age and capacity? | | |
| Is it possible that the study will involve greater than minimal privacy risks, which could induce stress to research participants, such as political behaviour, illegal and sexual conduct, drug or alcohol use? | | |
| Will the study cause psychological stress/pain/discomfort?<br>If YES, please state the precautions taken to minimize such stress/pain/discomfort/risk :<br>____________________________________ | | |

| | | |
|---|---|---|
| Are any of these participants from a minority/culturally identifiable/disadvantaged group?<br>(e.g. Orang ASIO<br><br>Please specify: Click here to enter text. | | |

i. If any of the responses above is yes, describe potential risk/conflict of interest of the study and provide a plan to mitigate the risk/conflict of interest.

The intervention will be in term of education program. This program will include educational booklets and educational classes. It will be carried out in a singleblind trial. The education program has no anticipated risk.

PART D: Privacy and Confidentiality

13. Describe how you will preserve participant's confidentiality as you collect and analyse the data and when you report the result

All participants will know that this study is confidential and all information provided concerning their identity will remain private. When presenting the study results or publishing, their identity will not be revealed without their expressed consent. All questionnaires will be coded and information that can identify them will be removed. Only the study investigators will be able to link the code with them. The information will be kept for a maximum period of 6 years from the end of data analysis and will be placed in a sealed envelope. After a lapse of such period, these forms will be destroyed through a shredding machine located at UM and will be witnessed by my supervisor and an academic staff. The soft copy and record data will be stored in a computer with a username and password to gain authorized access only by the main researcher and my supervisor. Confidentiality will be maintained by coding the name. Otherwise, a soft copy will be deleted and we will be doing computer storage reformat to destroy the database after a maximum period of 6 years, at the same time with questionnaire and database of hard copy.

14. Existing data (if you are using existing records containing personal data).

i. Please state the source of the data.

No existing data will be used. This study will generate primary data which is the survey questionnaire.

il. Are the data sensitive? (e.g. sexual preference, health status, criminal activity

| | |
|---|---|
| | YES |
| | NO |

iii. Please provide full details of types of personal data to be used:

15. Data record.

i. Describe briefly how the research data will be recorded, for example, audiotape, videotape or written notes.

Written notes the questionnaire will be used to collect the data and the data will enter directly to SPSS software. The questionnaire consists of section A which is general information (Socio-

Demographic Characteristics and Anthropometric Characteristics) and Section B which is consist of 7 parts (First: Knowledge, Attitude, and Practice on Nutrition and physical Activity questionnaire (KAP-Q), Second: Physical Activity Questionnaire for Older Children (PAQC), Third: Adolescents Sedentary Activity Questionnaires (ASAQ), Fourth: Food Frequency Questionnaire to assess food intake of obese and overweight children and adolescents, Fifth 24hour eating recall for three day, Sixth: Eating Attitudes Test (EAT-26) and Seventh: HBM Questionnaire.

ii. Describe what you will do with the recorded data once it has been analysed.

Data will be analysed using SPSS software and the results of this study will be published. When publishing or presenting the study results, participants' identity will not be revealed without their expressed consent. All questionnaires will be coded and information that can identify them will be removed. Only the study investigators will be able to link the code with them. The

information will be kept for a maximum period of 6 years from the end of data analysis and will be placed in a sealed envelope. After a lapse of such period, these forms will be destroyed through a shredding machine located at UM and will be witnessed by my supervisor and an academic staff. The soft copy and record data will be stored in a computer with a username and password to gain authorized access only by the main researcher and my supervisor. Confidentiality will be maintained by code the name. Otherwise, a soft copy will be deleted and we will be doing windows setup in computer to destroy the database after a maximum period of 6 years, at the same time with questionnaire and database of hard copy.

iii. Specify who apart from yourself will have access to the research data.

Only the study investigators will be able to access to the research data.

I-Dr. Nik Daliana Nik Farid

2-Dr. Mohmad Shafiq Bin Azanan

iv. Details who will own the data and the results of your research.

UM will owns the research.

## PART D: Conflict of Interest

16. Do any of the researchers have any potential conflicts of interest?

None of the researchers had any potential conflicts of interest.

## PART E: ATTACHMENTS

Please tick the boxes- which of the following documents are enclosed.

| | |
|---|---|
| | Questionnaire/ interview script |
| | Participant Information Sheet |
| | Informed Consent Form |

| | |
|---|---|
| | Others : Please state :<br>l- Consent form (Arabic )_<br>2- Participant Information Sheet(Arabic) 3-<br>Schools names and addresses.<br>4- Questionnaire (Arabic) |

PART D: Declaration

"In making this application, I certify that I have read and understand the Code of Ethics and University of Malaya Manual for Responsible Research and I will comply with the ethical principles of these documents. I will submit, as appropriate, a report for amendment of an approved project, if there are significant changes to my research or if there is an adverse incident".

Signature

Name

Date

Signature of Supervisor:

Name

Stamp

I hereby endorse that this applicant is appropriately qualified in the research area involved to conduct the proposed research project and is capable of undertaking this research study in a safe and ethical manner.

Signature

(Dean of Faculty [Head of Department)

Name

Date

Stamp

# CONSENT FORM

**Title of research:**

THE EFFECTIVENESS OF HEALTH LITERACY INTERVENTION ON PHYSICAL ACTIVITY AND HEALTHY DIET AMONG MIDDLE EASTERN ADOLESCENTS IN MALAYSIA

I herewith confirm that I have met the requirement of age and am capable of acting on behalf of myself /* as a parent/legal guardian as follows*:*

1. I understand the nature and scope of the research beingundertaken.
2. All my questions relating to this research and my participation therein have been answered to my satisfaction.
3. I voluntarily agree to take part in this research, to follow the study procedures and to provide all necessary information to the investigators as requested.
4. I may at any time choose to withdraw from this research without givingreasons.
5. I have received a copy of the Subjects Information Sheet and Consent Form.
6. Except for damages resulting from negligent or malicious conduct of the researcher(s), I hereby release and discharge University of Malaya and all participating researchers from all liability associated with, arising out of, or related to my participation and agree to hold them harmless from any harm or loss that may be incurred by me due to my participation in theresearch.
7. I have read and understood all the terms and conditions of my participation in the research.

I have read the statements above, understand the same, and voluntarily sign this form.
Dated :______day______month_______year

Name of participants (18 years & above): ____________________________________________________

IC/Passport number: ______________________________________

Signature:__________________________ Date:________________________

***To be filled by parents/legal guardians for participants aged below 18 years***

Name of parent/legal guardian: _____________________________________________________________

IC/Passport number Parent/legal guardian: _____________________________________

Signature of parent/legal guardian:_________________ Date:______________________

Ralationship of the guardian to the participant:__________________________________________

Name of researcher:_____________________________IC/Passport number: _____________________

Signature of researcher: __________________________

# نموذج الموافقة

اسم البحث:فعالية التدخل في معرفة القراءة والكتابة الصحية في النشاط البدني والنظام الغذائي الصحي بين المراهقين الشرق األوسط في ماليزيا.اؤكد أنني استوفيت شرط العمر وأنني قادر على التصرف نيابة عني / * بصفتي ولي أمر / وصي قانوني على

النحو التالي:

1. أفهم طبيعة ونطاق البحث الجاري.

2. تم الرد على جميع أسئلتي المتعلقة بهذا البحث ومشاركتي فيه بشكل كلي.

3. أوافق طواعية على المشاركة في هذا البحث واتباع إجراءات الدراسة وتقديم كل المعلومات الالزمة للباحثين على النحوالمطلوب.

4. قد أختار في أي وقت االنسحاب من هذا البحث دون إبداء األسباب.

5. لقد تلقيت نسخة من ورقة معلومات المشارك ونموذج الموافقة.

6. فيما عدا األضرار الناتجة عن إهمال أو سلوك مؤٍذ للباحث )الباحثين( ، أنا بموجب هذا إعفي وإبرء ذمة جامعة ماااليا وجميع

الباحثين المشاركين من كل مسئولية مرتبطة أو ناشئة عن أو مرتبطة بمشاركتي وأوافق على إبعادهم عن أي ضرر أو خسارة قد

أتحملها بسبب مشاركتي في البحث.

7. لقد قرأت وفهمت جميع شروط وأحكام مشاركتي في البحث.

لقد قرأت البيانات الواردة أعاله ، وفهمت األمر نفسه ، وأوقعت طواعية على هذا النموذج. السنة التاريخ اليوم
: التاريخ

:( __ فوق فما سنة )18 المشارك اسم
الجواز او الهوية رقم التوقيع :التاريخ :

يجب ملؤها من قبل الوالدين / األوصياء القانونيين للمشاركين الذين تقل أعمارهم عن 18 عاًما

القانون الوصي / الوالد اسم
القانون الوصي / للوالد الجواز او الهوية رقم

القانون الوصي / الوالد توقيع :التاريخ :

القانوني والوصي المشارك بين القرابة صلة

الباحث :الجواز او الهوية رقم : الباحث توقيع

## PARTICIPANT INFORMATION SHEET(parents)

Your child is being invited to participate in this research study. Before you decide it is important for you to understand why the research is being done and what it will involve. Please take time to read the following information carefully and discuss it with others if you wish. Ask us if there is anything that is not clear or if you would like more information. Take time to decide whether or not you wish to take part.

**Title of research:**

THE EFFECTIVENESS OF HEALTH LITERACY INTERVENTION ON PHYSICAL ACTIVITY AND HEALTHY DIET AMONG MIDDLE EASTERN ADOLESCENTS IN MALAYSIA

**Introduction:** Childhood obesity is one of the most serious public health challenges of the 21st century globally. Overweight and obese children are likely to stay obese into adulthood and more likely to develop non-communicable diseases like diabetes and cardiovascular diseases at a younger age. According to the Centers for Disease Control and Prevention (CDC), obesity prevalence among adolescents aged 12 to 19 years old has increased globally from 5% in 1980 to over 21% in 2012. An estimated 17.6 million children worldwide are overweight. In Malaysia, Middle Eastern adolescents are going through changes in living status, yet few studies show that overweight and obesity are prevalent among Arabic secondary school students. The researcher will apply to an educational program. The educational program will be designed to improve knowledge and promote lifestyle adherence in terms of healthy diet behaviour and physical activity.

**Purpose of the study:** To determine the effects of intervention programs on physical activity and healthy diet behaviour among Middle Eastern adolescent students in Arabic schools in Malaysia.

**Study Procedure**: Participation in this research is voluntary. Whatever you decide, you will not lose any benefits to which you are otherwise entitled. During the initial part of the study, you will be required to fill out a set of questionnaires. the questionnaire will be used to collect the data and the data will be deidentified before entered into a statistical analysis software analysed by SPSS. The questionnaire consists of section A which is general information (Socio-Demographic Characteristics) along with your weight, height, waist Circumference, and abdominal obesity status. Section B consists of 7 questionnaires. Then you will receive a booklet (on a healthy diet and physical activity). You will have to read it within one week and you will participate in six sessions during 6 weeks. The sessions will be conducted in school and each session will take 45 minutes (it consists of 15 minutes for related instruction, 15 minutes for group discussion and 15 minutes for role-playing). During the sessions, we will discuss a healthy diet and physical activity. After 6 weeks the questionnaires will be administered again.

**Participation in the Study:**

As previously mentioned, some behaviours in adolescence contribute to about one-third of adulthood diseases. Therefore, it is crucial to increase the awareness of the risks of those behaviours among middle eastern adolescents.

You are invited to participate because you are from the Middle East, aged 13 to 14 years and you and your parents agree to participate in the study. Your participation is voluntary. It is possible for you to withdraw from the research project and it is ok for you to do that at any time.

**The benefit to participants:** The information that is collected during this study will give us a better understanding of unhealthy habits and behaviours and the effectiveness of our programme in improving them into healthy ones. Consequently, it will help us to provide a more comprehensive programme to be applied to your colleagues in the future.

**Risk to participants:** The only possible drawback can be your time to participate in the class.

**Confidentiality**: No video or audio taping will be involved. All of the information collected is confidential and all data will be pooled and published in aggregate form only with no reference to a specific individual. You will be identified by an ID number so no personal identification will be obtained. Only the researcher will have access to the information you provide. The data will be stored in the researcher's office on a secure server. Hence, the data from each individual will remain confidential. By signing on the consent form, it will authorize the review, analysis and use of the data arising from this study. If you have any questions about the study or your rights, please contact the researcher using the contact information provided.

**Contact for Further Information**

For further information, please contact

**HANAN HASAN AL-HARONI**

**Telephone: 0172855427**

**Complaints:**

If you have any concerns or questions about the research project, which you do not wish to discuss with the researcher listed above, then you may contact:

**Reviewing REC:** University of Malaya Research Ethics Committee (UMREC)

**Telephone:** 03-79677022 (ext : 2369)

**Email:** umrec@um.edu.my

**Mailing address :**Pusat Perkhidmatan Penyelidikan (PPP) Level 2, Kompleks Pengurusan Penyelidikan dan Inovasi (KPPI) University of Malaya 3 50603 Kuala Lumpur, Malaysia

**NB**: You will be given a copy of the information sheet and a signed consent form to keep.

# ورقة معلومات المشارك

ابنك/ابنتك/اوصيائك مدعو للمشاركة في دراسة بحثية. قبل أن تقرر أنه من المهم بالنسبة لك أن تفهم سبب إجراء البحث وما الذي سيتضمنه. يرجى تخصيص بعض الوقت لقراءة المعلومات التالية بعناية ومناقشتها مع اآلخرين إذا كنت ترغب في ذلك. اسألنا إذا كان هناك أي شيء غير واضح أو إذا كنت ترغب في مزيد من المعلومات. خذ وقتك لتقرير ما إذا كنت ترغب في المشاركة أم ال

اسم البحث: فعالية التدخل في معرفة الوعي الصحي للنشاط البدني والنظام الغذائي الصحي بين مراهقين الشرق األوسط في ماليزيا.

المقدمة: تعتبر السمنة لدى األطفال من أخطر تحديات الصحة العامة في القرن الحادي والعشرين على مستوى العالم. من المرجح

أن يظل األطفال الذين يعانون من زيادة الوزن والسمنة يعانون من السمنة المفرطة في مرحلة المراهقة عرضة لإلصابة بأمراض غير

معدية مثل مرض السكري وأمراض القلب واألوعية الدموية في سن أصغر. وفق ًا لمراكز السيطرة على األمراض والوقاية منها

)CDC( ، فقد زاد انتشار السمنة بين المراهقين الذين تتراوح أعمارهم بين 12 و 19 عا ًما على مستوى العالم من 5٪ في عام

1980 إلى أكثر من 21٪ في عام 2012. ويقدر أن 17.6 مليون طفل في جميع أنحاء العالم يعانون من زيادة الوزن. وفي ماليزيا ، يمر المراهقون من الشرق األوسط بتغييرات في الحالة المعيشية ، و بعض من هذة الدراسات تظهر أن زيادة الوزن والسمنة منتشرة بين طالب المدارس الثانوية العربية في ماليزيا. يقدم الباحث في هذا البحث برنامج تعليمي للمراهقين . بحيث سيتم تصميم البرنامج التعليمي لتحسين المعرفة وتعزيز االلتزام بنمط الحياة الصحي فيما يتعلق بالسلوك الغذائي السوى والنشاط البدني.

الغرض من الدراسة: لتحديد تأثير برامج التدخل التعليمي على النشاط البدني والسلوك الغذائي الصحي بين طالب الشرق األوسط المراهقين في المدارس العربية في ماليزيا.

إجراءات الدراسة البحثية: المشاركة في هذا البحث تطوعية. أيا كان ما تقرره ، فلن تفقد أي مزايا يحق لك الحصول عليها .

خالل الجزء األول من الدراسة ، سي ُطلب منك ملء مجموعة من االستبيانات. سيتم استخدام االستبيان لجمع البيانات وسيتم تحديد

البيانات قبل إدخالها في برنامج التحليل اإلحصائي الذي تم تحليله بواسطة SPSS. يتكون االستبيان من القسم أ وهو عبارة عن

معلومات عامة )الخصائص االجتماعية والديموغرافية( وكذلك وزنك وطولك ومحيط الخصر وقياس حالة السمنة في منطقة البطن. القسم ب يتكون من 7 استبيانات. ثم ستحصل على كتيب )عن النظام غذائي الصحي والنشاط البدني(. سيتعين عليك قراءته في غضون أسبوع واحد وستشارك في ست جلسات توعوية خالل 6 أسابيع. سيتم إجراء الجلسات في المدرسة وستستغرق كل جلسة 45 دقيقة )تتكون من 15 دقيقة للتعليمات ذات الصلة ، و 15 دقيقة للمناقشة الجماعية و 15 دقيقة لتمثيل األدوار(. خالل الجلسات ،

سنناقش النظام الغذائي الصحي والنشاط البدني. بعد 6 أسابيع ستتم إعادة االستبيانات مرة أخرى.

المشاركة في الدراسة: كما ذكرنا سابق ًا ، تساهم بعض السلوكيات في مرحلة المراهقة في حوالي ثلث أمراض البلوغ. لذلك

من الضروري زيادة الوعي بمخاطر هذه السلوكيات بين المراهقين في الشرق األوسط. أنت مدعو للمشاركة ألنك من منطقة الشرق

األوسط ، وتتراوح عمرك بين 13 و 14 عا ًما وتوافق أنت ووالديك على المشاركة في الدراسة. مشاركتك طوعية. من الممكن أن

تنسحب من مشروع البحث وال بأس في أن تفعل ذلك في أي وقت.

الفائدة المرجوة للمشاركين: ستمنحنا المعلومات التي سيتم جمعها خالل هذه الدراسة فه ًما أفضل للعادات والسلوكيات غير

الصحية وفعالية برنامجنا في تحسينها إلى سلوكيات صحية. وبالتالي ، سيساعدنا ذلك في توفير برنامج أكثر شموال ً لتطبيقه على
زمالئك في المستقبل.
المخاطر على المشاركين: قد يكون العيب الوحيد المحتمل هو وقتك للمشاركة في الفصل.
الخصوصية: لن يتم تضمين أي فيديو أو تسجيل صوتي. جميع المعلومات سيتم جمعها في سرية وسيتم تجميع جميع البيانات

ونشرها في شكل إجمالي فقط دون اإلشارة إلى فرد معين. سيتم تحديد هويتك برقم هوية ، لذلك لن يتم الحصول على هوية شخصية.

سيتمكن الباحث فقط من الوصول إلى المعلومات التي تقدمها. سيتم تخزين البيانات في مكتب الباحث على سيرفر آمن. وبالتالي ، ستبقى البيانات
من كل فرد سرية. من خالل التوقيع على استمارة الموافقة ، ستسمح بمراجعة وتحليل واستخدام البيانات الناشئة عن هذه
الدراسة. إذا كانت لديك أي أسئلة حول الدراسة او المتعلقة بحقوقك ، فيرجى االتصال بالباحث باستخدام معلومات االتصال

المقدمة.

للحصول على مزيد من المعلومات:

لمعلومات أكثر، يرجى االتصال على الباحثة

حنان حسن الهاروني

هاتف: 0172855427

في حالة الشكاوي: إذا كانت لديك أية مخاوف أو أسئلة حول مشروع البحث ، وال ترغب في مناقشتها مع الباحث المذكور أعاله ،

فيمكنك االتصال بـ:

**Reviewing REC:** University of Malaya Research Ethics Committee (UMREC)

**Telephone:** 03-79677022 (ext : 2369)

**Email:** umrec@um.edu.my

**Mailing address :**Pusat Perkhidmatan Penyelidikan (PPP) Level 2, Kompleks Pengurusan Penyelidikan dan Inovasi (KPPI) University of Malaya 3 50603 Kuala Lumpur, Malaysia

**مالحظة:** ستحصل على نسخة من ورقة المعلومات واستمارة موافقة موقعة لالحتفاظ بها

## PARTICIPANT INFORMATION SHEET(adolesents)

You are invited to take part in a research study. Before you decide it is important for you to understand why the research is being done and what it will involve. Please take time to read the following information carefully and discuss it with others if you wish. Ask us if there is anything that is not clear or if you would like more information. Take time to decide whether or not you wish to take part.

**Title of research:**

THE EFFECTIVENESS OF HEALTH LITERACY INTERVENTION ON PHYSICAL ACTIVITY AND HEALTHY DIET AMONG MIDDLE EASTERN ADOLESCENTS IN MALAYSIA

**Introduction:** Childhood obesity is one of the most serious public health challenges of the 21st century globally. obesity prevalence among adolescents aged 12 to 19 years old has increased globally from 5% in 1980 to over 21% in 2012. An estimated 17.6 million children worldwide are overweight. In Malaysia, Middle Eastern adolescents are going through changes in living status, yet few studies show that overweight and obesity are prevalent among Arabic secondary school students. The researcher will apply to an educational program. The educational program will be designed to improve knowledge and promote lifestyle adherence in terms of healthy diet behaviour and physical activity.

**Purpose of the study:** To determine the effects of intervention programs on physical activity and healthy diet behaviour among Middle Eastern adolescent students in Arabic schools in Malaysia.

**What you have to do?**: Participation in this research is voluntary. Whatever you decide, you will not lose any benefits to which you are otherwise entitled. During the initial part of the study, you will be required to fill out a set of questionnaires. the questionnaire will be used to collect the data and the data will be deidentified before entered into a statistical analysis software analysed by SPSS. Then you will receive a booklet (on a healthy diet and physical activity). You will have to read it within one week and you will participate in six sessions during 6 weeks. The sessions will be conducted in school and each session will take 45 minutes (it consists of 15 minutes for related instruction, 15 minutes for group discussion and 15 minutes for role-playing). During the sessions, we will discuss a healthy diet and physical activity. After 6 weeks the questionnaires will be administered again.

**Who should Participation in the Study?**

You are invited to participate because you are from the Middle East, aged 13 to 14 years and you and your parents agree to participate in the study. Your participation is voluntary. It is possible for you to withdraw from the research project and it is ok for you to do that at any time.

**What will be the benefit to participants?** The information that is collected during this study will give us a better understanding of unhealthy habits and behaviours and the effectiveness of our programme in improving them into healthy ones. Consequently, it will help us to provide a more comprehensive programme to be applied to your colleagues in the future.

**What will be the risk to participants?** The only possible drawback can be your time to participate in the class.

**Confidentiality**: No video or audio taping will be involved. All of the information collected is confidential and all data will be pooled and published in aggregate form only with no reference to a specific individual. You will be identified by an ID number so no personal identification will be obtained. Only the researcher will have access to the information you provide. The data will be stored in the researcher's office on a secure server. Hence, the data from each individual will remain confidential. By signing on the consent form, it will authorize the review, analysis and use of the data arising from this study. If you have any questions about the study or your rights, please contact the researcher using the contact information provided.

**Contact for Further Information**

For further information, please contact

**HANAN HASAN AL-HARONI**

**Telephone: 0172855427**

**Complaints:**

If you have any concerns or questions about the research project, which you do not wish to discuss with the researcher listed above, then you may contact:

**Reviewing REC:** University of Malaya Research Ethics Committee (UMREC)

**Telephone:** 03-79677022 (ext : 2369)

**Email:** umrec@um.edu.my

**Mailing address :**Pusat Perkhidmatan Penyelidikan (PPP) Level 2, Kompleks Pengurusan Penyelidikan dan Inovasi (KPPI) University of Malaya 3 50603 Kuala Lumpur, Malaysia

**NB**: You will be given a copy of the information sheet and a signed consent form to keep.

# ورقة معلومات المشارك ( للمراهقين)

انت مدعو للمشاركة في دراسة بحثية. قبل أن تقرر أنه من المهم بالنسبة لك أن تفهم سبب إجراء البحث وما الذي سيتضمنه. يرجى تخصيص بعض الوقت لقراءة المعلومات التالية بعناية ومناقشتها مع اآلخرين إذا كنت ترغب في ذلك. اسألنا إذا كان هناك أي شيءغير واضح أو إذا كنت ترغب في مزيد من المعلومات. خذ وقتك لتقرير ما إذا كنت ترغب في المشاركة أم ال

اسم البحث: فعالية التدخل في معرفة الوعي الصحي للنشاط البدني والنظام الغذائي الصحي بين مراهقين الشرق األوسط في ماليزيا.

المقدمة: تعتبر السمنة لدى األطفال من أخطر تحديات الصحة العامة في القرن الحادي والعشرين على مستوى العالم. فقد زاد

انتشار السمنة بين المراهقين الذين تتراوح أعمارهم بين 12 و 19 عاًما على مستوى العالم من 5٪ في عام 1980 إلى أكثر

من 21٪ في عام 2012. ويقدر أن 17.6 مليون طفل في جميع أنحاء العالم يعانون من زيادة الوزن. وفي ماليزيا ، يمر المراهقون

من الشرق األوسط بتغييرات في الحالة المعيشية ، و بعض من هذة الدراسات تظهر أن زيادة الوزن والسمنة منتشرة بين طالب

المدارس الثانوية العربية في ماليزيا. يقدم الباحث في هذا البحث برنامج تعليمي للمراهقين . بحيث سيتم تصميم البرنامج التعليمي

لتحسين المعرفة وتعزيز االلتزام بنمط الحياة الصحي فيما يتعلق بالسلوك الغذائي السوى والنشاط البدني.

الهدف من الدراسة: لتحديد تأثير برامج التدخل التعليمي على النشاط البدني والسلوك الغذائي الصحي بين طالب الشرق األوسط

المراهقين في المدارس العربية في ماليزيا.

ماهي إجراءات الدراسة البحثية؟ المشاركة في هذا البحث تطوعية. أيا كان ما تقرره ، فلن تفقد أي مزايا يحق لك الحصول عليها . ُطلب منك ملء مجموعة من االستبيانات. سيتم استخدام االستبيان لجمع البيانات خالل الجزء األول من الدراسة ، سي وسيتم تحديد

البيانات قبل إدخالها في برنامج التحليل اإلحصائي الذي تم تحليله بواسطة SPSS. ثم ستحصل على كتيب )عن النظام غذائي

الصحي والنشاط البدني(. سيتعين عليك قراءته في غضون أسبوع واحد وستشارك في ست جلسات توعوية خالل 6 أسابيع. سيتم إجراء الجلسات في المدرسة وستستغرق كل جلسة 45 دقيقة )تتكون من 15 دقيقة للتعليمات ذات الصلة ، و 15 دقيقة للمناقشة الجماعية و 15 دقيقة لتمثيل األدوار(. خالل الجلسات ، سنناقش النظام الغذائي الصحي والنشاط البدني. بعد 6 أسابيع ستتم إعادة االستبيانات مرة أخرى.

لماذا المشاركة في الدراسة؟ أنت مدعو للمشاركة ألنك من منطقة الشرق األوسط ، وتتراوح عمرك بين 13 و 14 عا ًما وتوافق

أنت ووالديك على المشاركة في الدراسة. مشاركتك طوعية. من الممكن أن تنسحب من مشروع البحث وال بأس في أن تفعل ذلك في

أي وقت.

ما الفائدة للمشاركة في الدراسة؟ ستمنحنا المعلومات التي سيتم جمعها خالل هذه الدراسة فهما ً أفضل للعادات والسلوكيات غير

الصحية وفعالية برنامجنا في تحسينها إلى سلوكيات صحية. وبالتالي ، سيساعدنا ذلك في توفير برنامج أكثر شموال ً لتطبيقه على زمالئك في المستقبل.

ماهي المخاطر على المشاركين في الدراسة؟ قد يكون العيب الوحيد المحتمل هو وقتك للمشاركة في الفصل.

الخصوصية: لن يتم تضمين أي فيديو أو تسجيل صوتي. جميع المعلومات سيتم جمعها في سرية وسيتم تجميع جميع البيانات

ونشرها في شكل إجمالي فقط دون اإلشارة إلى فرد معين. سيتم تحديد هويتك برقم هوية ، لذلك لن يتم الحصول على هوية شخصية.

سيتمكن الباحث فقط من الوصول إلى المعلومات التي تقدمها. سيتم تخزين البيانات في مكتب الباحث على سيرفر آمن. وبالتالي ، ستبقى البيانات من كل فرد سرية. من خالل التوقيع على استمارة الموافقة ، ستسمح بمراجعة وتحليل واستخدام البيانات الناشئة عن هذه الدراسة. إذا كانت لديك أي أسئلة حول الدراسة او المتعلقة بحقوقك ، فيرجى االتصال بالباحث باستخدام معلومات االتصال

المقدمة.
للحصول على مزيد من المعلومات:
للحصول على معلومات أكثر، يرجى االتصال على الباحثة

حنان حسن الهاروني

هاتف0172855427 :

في حالة كان لديك شكوى: إذا كانت لديك أية مخاوف أو أسئلة حول مشروع البحث ، وال ترغب في مناقشتها مع الباحث المذكور

أعاله ، نيمكنك االتصال بـ: **Reviewing REC:** University of Malaya Research Ethics Committee (UMREC)

**Telephone:** 03-79677022 (ext : 2369)

**Email:** umrec@um.edu.my

**Mailing address :**Pusat Perkhidmatan Penyelidikan (PPP) Level 2, Kompleks Pengurusan Penyelidikan dan Inovasi (KPPI) University of Malaya 3 50603 Kuala Lumpur, Malaysia

ملاحظة: ستحصل على نسخة من ورقة المعلومات واستمارة موافقة موقعة لالحتفاظ بها.

**Appendix 1: Questionnaires**

**Section A**

**First Part: Socio-Demographic Characteristics**

| | | | |
|---|---|---|---|
| **Age** | ____________________Years | | |
| **Date of birth** | Day | Month | Year |
| **School name** | | | |
| **Grade** | 7th ☐ | 8th ☐ | |
| **Number of siblings** | | | |
| **Number of household** | | | |
| **Mother's education** | Intermediate school or lower ☐ | High school ☐ | Undergraduate degree or higher ☐ |
| **Father's education** | Intermediate school or lower ☐ | High school ☐ | Undergraduate degree or higher ☐[3] |
| **Monthly income (RM)** | Less than 5000 ☐ | 5000-14,999 ☐ | More or equal 15,000 High ☐ |

**Second Part: Anthropometric Characteristics**

| | |
|---|---|
| **Weight (kg)** | |
| **Height (cm)** | |
| **Z_score** | |
| **Waist Circumference (cm)** | |
| **Abdominal obesity status (cm)** | |

**Section B: Questionnaire**

**1: Knowledge, Attitude, and Practice on Nutrition and physical Activity (KAP-Q)**

| **No.** | Knowledge (1-30): please choose only one answer that you think is best for you. There is no right or wrong answer in this part |
|---|---|
| 1. | Body image dissatisfaction affects body health negatively<br>a) True<br>b) False<br>c) I don’t know |
| 2. | Vegetables and fruits are best sources for fibre<br>a) True<br>b) False<br>c) I don't know |
| 3. | Everyone can practice physical activity regularly regardless health status<br>a) True<br>b) False<br>c) I don't know |
| 4. | Skipping is classified as low physical activity<br>a) True<br>b) False<br>c) I don't know |
| 5. | Regular physical activity is important for all ages<br>a) True<br>b) False<br>c) I don't know |
| 6. | Food label can show less information about calories<br>a) True<br>b) False<br>c) I don’t know |
| 7. | Adolescents (13-14) years need to eat healthy food regardless of practicing physical activity<br>a) True<br>b) False<br>c) I don’t know |
| 8. | Adolescents (13-14) years can spend 4 hours per day on screen time such as iPad, mobile phone, or other devices<br>a) True<br>b) False<br>c) I don't know |
| 9. | One of activities which is not considered sedentary behavior is sitting while playing music<br>a) True<br>b) False<br>c) I don't know |
| 10. | Adolescences (13-14) years can skip one of the main meals each day<br>a) True<br>b) False<br>I don’t know |

| | |
|---|---|
| 11. | Cycling is classified as an aerobic physical activity<br>a) True<br>b) False<br>c) I don't know |
| 12. | Body Mass Index (BMI) = weight(kg) / length (m)2<br>a) True<br>b) False<br>c) I don’t know |
| 13. | When the amount of food calories taken is 2000, and energy expenditure is 1800 calories. In this situation weight is loss<br>a) True<br>b) False<br>c) I don't know |
| 14. | Physical activity is a movement produced by big muscles contractions which require energy consumption<br>a) True<br>b) False<br>c) I don’t know |
| 15. | You need to measure weight and height, and body mass index (BMI) to monitor your growth<br>a) True<br>b) False<br>c) I don’t know |
| 16. | Fat food can increase obesity<br>a) True<br>b) False<br>I don't know |
| 17. | Soft drinks have large amount of minerals<br>a) True<br>b) False<br>c) I don’t know |
| 18. | Obesity is not increasing risks of diabetes type 2<br>a) True<br>b) False<br>c) I don't know |
| 19. | The time of sedentary activities should be 4 hours per day<br>a) True<br>b) False<br>c) I don't know |
| 20. | It is better to practice one type of physical activity every day<br>a) True<br>b) False<br>c) I don’t know |
| 21. | Fatima can replace sedentary lifestyle by playing computer games<br>a) True<br>b) False<br>c) I don’t know |
| 22. | Unsaturated fat is good if it presents in food<br>a) True<br>b) False<br>c) I don’t know |

| | |
|---|---|
| 23. | Chatting with friends while sitting at living room for an hour or more every day can improve your body fitness<br>a) True<br>b) False<br>c) I don’t know |
| 24. | Based on the Saudi Healthy Food Palm, taken 2 – 3 serving size of vegetables every day will makes you healthy<br>a) True<br>b) False<br>c) I don’t know |
| 25. | Warm up exercises are important before any other exercise<br>a) True<br>b) False<br>c) I don’t know |
| 26. | Walking has a vital role in achieving longevity of life<br>a) True<br>b) False<br>c) I don't know |
| 27. | Physical activity reduces stress<br>a) True<br>b) FalseI don’t know |
| 28. | Physical inactivity increases risk of type 2 diabetes<br>a) True<br>b) False<br>c) I don’t know |
| 29. | Ordering big size of food because of sale promotions is not a healthy eating practice<br>a) True<br>b) False<br>c) I don’t know |
| 30. | Drinking water before, during, and after walking is important for your body health<br>a) True<br>b) False<br>c) I don't know |

**Attitude part (1-22): please choose only one answer that you think is best for you. There is no right or wrong answer in this part**

| NO. | Statements | Strongly Disagree | Disagree | Sometimes | Agree | Strongly agree |
|---|---|---|---|---|---|---|
| 1 | I think eating healthy food is more important than doing physical activity for our bodies' health | | | | | |
| 2 | I think the way of cooking will not effect on my choice of food outside | | | | | |
| 3 | For my body health, I think skipping breakfast is not good | | | | | |
| 4 | I think eating healthy food is not important for growth | | | | | |
| 5 | I think I can increase my physical activity gradually in order to get health benefits | | | | | |
| 6 | I think helping my family with household chores is important to increase physical activity | | | | | |
| 7 | I like to spend more than 3 hours on mobile phone every day | | | | | |
| 8 | I should practice physical activity every day such as walking not more than 10 minutes | | | | | |
| 9 | I think obesity is not related to eating high amount of calories | | | | | |
| 10 | I think doing physical activity should be done outdoor | | | | | |
| 11 | I should follow the Healthy Food Palm to help me eat healthy food | | | | | |
| 12 | I think there is a relationship between healthy food and academic achievement | | | | | |
| 13 | I think sedentary behaviour not affect body weight | | | | | |
| 14 | I believe body health is affected by an imbalance between the quantity of food energy taken and the quantity of food energy consumption | | | | | |
| 15 | I believe what I eat every day will not affect my future body health | | | | | |

| | | | | | | |
|---|---|---|---|---|---|---|
| 16 | Starchy foods should be recommended in every main meal | | | | | |
| 17 | I think recommendations of physical activity help me to be an active student | | | | | |
| 18 | I think I can do moderate physical activity at least 60 minutes every day | | | | | |
| 19 | My perception of my body image cannot affect my health status | | | | | |
| 20 | I think that doing more physical activity can help me to prevent type 2 diabetes | | | | | |
| 21 | The amount of calories on food’s label does not influence my choice of food | | | | | |
| 22 | I think doing physical activity can help me to control my body weight | | | | | |

| Practice part (1- 21): please choose only one answer that you think is best for you. There is no right or wrong answer in this part | | | | | | |
|---|---|---|---|---|---|---|
| NO. | **How often do you** | Very frequencies | Often | Sometimes | Rarely | Never |
| 1 | Practice any type of physical activity in the suitable place and time | | | | | |
| 2 | Skip walking as a physical activity without your family or friends | | | | | |
| 3 | Get 4 hours or more in sedentary behaviour such as watching TV or chatting with friends every day | | | | | |
| 4 | Order fried food instead of grilled food | | | | | |
| 5 | Skip drinking water when you practice moderate physical activity for 60 minutes or more | | | | | |
| 6 | Skip reading the nutritional value of food labels when buying food | | | | | |
| 7 | Replace your time spend in front of screen such as your mobile phone or iPad by physical activity | | | | | |
| 8 | Skip main meals' time to reduce your weight | | | | | |
| 9 | Increase physical activity in every opportunity such as help your mother in chores | | | | | |
| 10 | Practice moderate physical activity for 60 minutes every day | | | | | |
| 11 | Skip daily physical activity | | | | | |
| 12 | Include a variety of vegetables and fruit in your meals every day | | | | | |
| 13 | Walk as a physical activity every day | | | | | |

| | | | | | | |
|---|---|---|---|---|---|---|
| 14 | Avoid eating increasing fat food when you are eating outside to prevent obesity | | | | | |
| 15 | Look in the mirror to check your body shape | | | | | |
| 16 | Order soft drinks when you eat outside | | | | | |
| 17 | Practice physical activity to maintain your health body | | | | | |
| 18 | Practice warm up before any exercise | | | | | |
| 19 | Check your body weight in order to maintain or to reach normal weight | | | | | |
| 20 | Replace low physical activity by moderate physical activity | | | | | |
| 21 | Skip drinking milk or dairy products in your dish every day | | | | | |

**Second: Physical Activity Questionnaire for Older Children (PAQ-C) after modified by experts from Saudi Arabia**

We are trying to find out about your level of physical activity from **the last 7 days** (in the last week). This includes sports or dance that make you sweat or make your legs feel tired, or games that make you breathe hard, like tag, skipping, running, climbing, and others.

**Remember:**
1. There are no right and wrong answers — this is not a test.
2. Please answer all the questions as honestly and accurately as you can — this is very important.

| **1-Physical activity in your spare time: Have you done any of the following activities in the past 7 days (last week)? If yes, how many times? (Mark only one circle per row.)** | **No** | **1-2** | **3-4** | **5-6** | **≥ 7 times** |
|---|---|---|---|---|---|
| a. Skipping | | | | | |
| b. Tag | | | | | |
| c. Game chairs | | | | | |
| d. Dancing with the Ring | | | | | |
| e. Pull the cord | | | | | |
| f. Ice Skating shoes | | | | | |
| g. Walking for exercise | | | | | |
| h. Hideaway | | | | | |
| i. Jogging or running | | | | | |
| j. Aerobics | | | | | |
| k. Swimming | | | | | |
| l. Dance | | | | | |
| m. Football | | | | | |
| n. Basketball | | | | | |
| o. Volleyball | | | | | |
| p. Bicycling | | | | | |
| q. Other | | | | | |

**2. In the last 7 days, during your physical education (PE) classes, how often were you very active (playing hard, running, jumping, throwing)? (Check one only.)**

a) I don't do PE .......................................................... ☐1
b) Hardly ever .............................................................. ☐2
c) Sometimes............................................................. ☐3
d) Quite often .............................................................. ☐4
e) Always ..................................................................... ☐5

**3. In the last 7 days, what did you do most of the time *at recess*? (Check one only.)**

a) Sat down (talking, reading, doing schoolwork).......... ☐1
b) Stood around or walked around ................................ ☐2
c) Ran or played a little bit .......................................... ☐3
d) Ran around and played quite a bit............................ ☐4
e) Ran and played hard most of the time ....................... ☐5

**4. In the last 7 days, what did you normally do *at lunch* (besides eating lunch)? (Checkone only.)**

a) Sat down (talking, reading, doing schoolwork)........... ☐1
b) Stood around or walked around ................................. ☐2
c) Ran or played a little bit ............................................ ☐3
d) Ran around and played quite a bit............................. ☐4
e) Ran and played hard most of the time ....................... ☐5

**5. In the last 7 days, on how many days *right after school*, did you do sports, dance, or play games in which you were very active? (Check one only.)**

a) None...................................................................... ☐1
b) 1 time last week ....................................................... ☐2
c) 2 or 3 times last week.............................................. ☐3
d) 4 times last week..................................................... ☐4
e) 5 times last week..................................................... ☐5

**6. In the last 7 days, on how many *evenings* did you do sports, dance, or play games in which you were very active? (Check one only.)**

a) None...................................................................... ☐1
b) 1-time last week ....................................................... ☐2
c) 2 or 3 times last week.............................................. ☐3
d) 4 or 5 last week ....................................................... ☐4
e) 6 or 7 times last week.............................................. ☐5

**7. *On the last weekend*, how many times did you do sports, dance, or play games in which you were very active? (Check one only.)**

a) None...................................................................... ☐1
b) 1 time ...................................................................... ☐2
c) 2 — 3 times............................................................. ☐3
d) 4 — 5 times............................................................. ☐4
e) 6 or more times ........................................................ ☐5

**8. Which *one* of the following describes you best for the last 7 days? Read *all five* statements before deciding on the *one* answer that describes you.** All or most of my free time was spent doing things that involve little

a) physical effort ........................................................................................................☐[1]
b) I sometimes (1 — 2 times last week) did physical things in my free time
c) (e.g. played sports, went running, swimming, bike riding, did aerobics)................ ☐[2]
d) I often (3 — 4 times last week) did physical things in my free time ....................... ☐[3]
e) I quite often (5 — 6 times last week) did physical things in my free time .............. ☐[4]
f) I very often (7 or more times last week) did physical things in my free time .........☐[5]

**9. Mark how often you did physical activity (like playing sports, games, doing dance, or any other physical activity) for each day last week.**

| | None | Little bit | Medium | Often | Very often |
|---|---|---|---|---|---|
| **Sunday** | | | | | |
| **Monday** | | | | | |
| **Tuesday** | | | | | |
| **Wednesday** | | | | | |
| **Thursday** | | | | | |
| **Friday** | | | | | |
| **Saturday** | | | | | |

**10. Were you sick last week, or did anything prevent you from doing your normal physical activities? (Check one.)**

a) Yes.......................................................... ☐[1]
b) No.......................................................... ☐[2]

If Yes, what prevented you? ___________________________________

## Third: Adolescents Sedentary Activity Questionnaires (ASAQ)

**Sedentary Activities**

NOW, SOME QUESTIONS ABOUT ACTIVITIES YOU DO SITTING DOWN
Overall, you have about 8 hours of free time before and after school, some of which is spent sitting. You may do multiple activities at the same time (play with smart phone in front of the TV), so please estimate how much time was spent on <u>*each*</u> activity within the total time spent doing both

Think about a normal *school week*, and write down how long you spend doing the following activities before and after school each day. Leave blank if you do not do that activity.

| Time / Activities | Sunday | | | Monday | | | Tuesday | | | Wednesday | | | Thursday | | |
|---|---|---|---|---|---|---|---|---|---|---|---|---|---|---|---|
| | **Hrs** | **Min** | | **Hrs** | **Min** | | **Hrs** | **Min** | | **Hrs** | **Min** | | **Hrs** | **Min** | |
| 1.Watching TV | | | | | | | | | | | | | | | |
| 2.Watching videos/DVDs | | | | | | | | | | | | | | | |
| 3.Using the computer for fun | | | | | | | | | | | | | | | |
| 4.Playing on smart phone or iPad | | | | | | | | | | | | | | | |
| 5.Playing computer or video games (Nintendo, Xbox, PlayStation, Wii) | | | | | | | | | | | | | | | |
| 6.Using the computer for doing homework | | | | | | | | | | | | | | | |
| 7.Doing homework not I computer | | | | | | | | | | | | | | | |
| 8.Reading for fun | | | | | | | | | | | | | | | |
| 9.Being tutored | | | | | | | | | | | | | | | |
| 10.Travel (car/bus/train) | | | | | | | | | | | | | | | |
| 11.Doing crafts or hobbies | | | | | | | | | | | | | | | |
| 12.Setting around chatting with friends/ on the phone/chilling | | | | | | | | | | | | | | | |
| 13.Playing/ practicing a musical instrument | | | | | | | | | | | | | | | |

Overall, you have about 16 hours of free time before and after school, some of which is spent sitting.

You may do multiple activities at the same time (play with smart phone in front of the TV), so please estimate how much time was spent on *each* activity within the total time spent doing both Think about a normal *school week*, and write down how long you spend doing the following activities before and after school each day. Leave blank if you do not do that activity.

| **Time** / **Activities** | **Friday** | | | **Saturday** | | |
|---|---|---|---|---|---|---|
| | **Hrs** | **Min** | | **Hrs** | **Min** | |
| 1.Watching TV | | | | | | |
| 2.Watching videos/DVDs | | | | | | |
| 3.Using the computer for fun | | | | | | |
| 4.Playing on smart phone or iPad | | | | | | |
| 5.Playing computer or video games (Nintendo, Xbox, PlayStation, Wii) | | | | | | |
| 6.Using the computer for doing homework | | | | | | |
| 7.Doing homework not I computer | | | | | | |
| 8.Reading for fun | | | | | | |
| 9.Being tutored | | | | | | |
| 10.Travel (car/bus/train) | | | | | | |
| 11.Doing crafts or hobbies | | | | | | |
| 12.Setting around chatting with friends/ on the phone/chilling | | | | | | |
| 13.Playing/ practicing a musical instrument | | | | | | |
| 14.Going to church or Saturday school | | | | | | |

**Fourth: Food Frequency Questionnaire to assess food intake of obese and overweight children and adolescents**

| No | Food name | Average size | Size if not average | | /day | | | | /week | | | /month | | |
|---|---|---|---|---|---|---|---|---|---|---|---|---|---|---|
| **Cereals and granis** | | | **Smaller** | **Larger** | Every | 2-3 times | 4-5 times | 6-7 times | 1-2 times | 3-4 times | 5-6 times | 1 times | 2-3 times | never |
| **1** | Bread | 1 slice | | | | | | | | | | | | |
| **2** | Macaroni | 1cup/250 ml | | | | | | | | | | | | |
| **3** | Corn flakes | 1cup/250 ml | | | | | | | | | | | | |
| **4** | Pop corn | One cup | | | | | | | | | | | | |
| **5** | Homos | 2 tbsp | | | | | | | | | | | | |
| **6** | Falafel | 2 piece medium | | | | | | | | | | | | |
| **7** | Biscuits | 1 Biscuits | | | | | | | | | | | | |
| **Fish, poultry and meat product** | | | **Smaller** | **Larger** | Every | 2-3 times | 4-5 times | 6-7 times | 1-2 times | 3-4 times | 5-6 times | 1 times | 2-3 times | never |
| **8** | Eegs | 1 egg | | | | | | | | | | | | |
| **9** | Sausage | 85 g | | | | | | | | | | | | |
| **10** | Fried fish | 270g/60 oz | | | | | | | | | | | | |
| **11** | Roast beef | 85 g | | | | | | | | | | | | |
| **12** | Tuna with oil | 1 can | | | | | | | | | | | | |
| **13** | Fried chicken | 85 g | | | | | | | | | | | | |
| **14** | Poached chicken | 85 g | | | | | | | | | | | | |
| **15** | Grilled chicken | 85 g | | | | | | | | | | | | |
| **16** | Chicken suop | 1 cup 250 ml | | | | | | | | | | | | |
| **17** | Boiled lamb | 85 g | | | | | | | | | | | | |
| **18** | Kofta kebab meat | 85 g | | | | | | | | | | | | |
| **Mixed dishes** | | | **Smaller** | **Larger** | Every | 2-3 times | 4-5 times | 6-7 times | 1-2 times | 3-4 times | 5-6 times | 1 times | 2-3 times | never |
| **19** | Chicken burger | 1 patty medium | | | | | | | | | | | | |
| **20** | Mutabiq | 85 g | | | | | | | | | | | | |
| **21** | Rice kabsa | ½ cup 17 ml | | | | | | | | | | | | |
| **22** | Cheese sandwiches | 1 patty medium | | | | | | | | | | | | |
| **23** | Egg sandwiches | 1 patty medium | | | | | | | | | | | | |
| **24** | Petza | 1 patty medium | | | | | | | | | | | | |
| **25** | Sandwiches meat | 1 slice | | | | | | | | | | | | |
| **26** | Samosas meat | Palm hand | | | | | | | | | | | | |
| **27** | Meat burger | 1 patty medium | | | | | | | | | | | | |
| **28** | Liver sandwiches | 1 patty medium | | | | | | | | | | | | |
| **29** | Chicken sandwiches | 1 patty medium | | | | | | | | | | | | |
| **30** | Sandwich mixed | 1 patty medium | | | | | | | | | | | | |
| **Dairy products** | | | **Smaller** | **Larger** | Every | 2-3 times | 4-5 times | 6-7 times | 1-2 times | 3-4 times | 5-6 times | 1 times | 2-3 times | never |
| 31 | Cream | 1 tbap/30ml | | | | | | | | | | | | |

| | | | | | | | | | | | | | | |
|---|---|---|---|---|---|---|---|---|---|---|---|---|---|---|
| | cheese | | | | | | | | | | | | | |
| 32 | Full- fat yogurt | 6 oz/170g | | | | | | | | | | | | |
| 33 | F- fat cow's milk | 1 cup 250ml | | | | | | | | | | | | |
| 34 | Low- fat cow's milk | 1 cup 250ml | | | | | | | | | | | | |
| 35 | Ice cream milk | ½ cup 17 ml | | | | | | | | | | | | |
| 36 | Milk chake | 1 cup 250ml | | | | | | | | | | | | |
| **Beverage** | | | **Smaller** | **Larger** | Every | 2-3 times | 4-5 times | 6-7 times | 1-2 times | 3-4 times | 5-6 times | 1 times | 2-3 times | never |
| **37** | Concentrated orange | ½ cup 175 ml | | | | | | | | | | | | |
| **38** | Canned u sweetened | ½ cup 17 ml | | | | | | | | | | | | |
| **39** | Minutes milk | 1 cup 250ml | | | | | | | | | | | | |
| **40** | Fresh orange juice | ½ cup 17 ml | | | | | | | | | | | | |
| **41** | Aerated cola drink | 1 cup 250ml | | | | | | | | | | | | |
| **42** | Apple juice | ½ cup 17 ml | | | | | | | | | | | | |
| **43** | Cappuccino | 1 cup 250ml | | | | | | | | | | | | |
| **44** | Instant coffee | 1 cup 250ml | | | | | | | | | | | | |
| **45** | Grape juice | ½ cup 17 ml | | | | | | | | | | | | |
| **Sweet and baked goods** | | | **Smaller** | **Larger** | Every | 2-3 times | 4-5 times | 6-7 times | 1-2 times | 3-4 times | 5-6 times | 1 times | 2-3 times | never |
| **46** | Pastries (fataer) | 1 medium patty | | | | | | | | | | | | |
| **47** | Honey | 1 tbsp | | | | | | | | | | | | |
| **48** | Mxid sweet | ½ cup | | | | | | | | | | | | |
| **49** | Sugar | 1 tbsp | | | | | | | | | | | | |
| **50** | Basbousa | 1 slice | | | | | | | | | | | | |
| **51** | Croissant fataye | 1 medium patty | | | | | | | | | | | | |
| **52** | Biscuit with dates | 1 piece | | | | | | | | | | | | |
| **53** | Baklava | 1 piece | | | | | | | | | | | | |
| **54** | Cake | 1 medium piece | | | | | | | | | | | | |
| **55** | Doughnuts jam | 1 medium piece | | | | | | | | | | | | |
| **56** | Cream desserts | ½ cup | | | | | | | | | | | | |
| **57** | Konafa cream | ½ cup | | | | | | | | | | | | |
| **58** | Sweet dumpling | 1 medium piece | | | | | | | | | | | | |
| **Fruits and vegetables** | | | **Smaller** | **Larger** | Every | 2-3 times | 4-5 times | 6-7 times | 1-2 times | 3-4 times | 5-6 times | 1 times | 2-3 times | never |
| **59** | Dates | 1 medium | | | | | | | | | | | | |
| **60** | Vegetable soup | 1 cup 250ml | | | | | | | | | | | | |
| **61** | Vegetable | ½ cup 17 ml | | | | | | | | | | | | |

| | | | | | | | | | | | | | | |
|---|---|---|---|---|---|---|---|---|---|---|---|---|---|---|
| | cooked | | | | | | | | | | | | | |
| **62** | Fava beans | ½ cup 17 ml | | | | | | | | | | | | |
| **63** | Nuts variety | ½ cup | | | | | | | | | | | | |
| **64** | Potato chips | 25 g baked | | | | | | | | | | | | |
| **65** | Boiled potatoes | 1 medium | | | | | | | | | | | | |
| **66** | Thyme | 1 tbsp | | | | | | | | | | | | |
| **67** | Black olive | 3 piece | | | | | | | | | | | | |
| **68** | Green salad mixed | ½ cup | | | | | | | | | | | | |
| **69** | Fresh tomatoes | 1 medium | | | | | | | | | | | | |
| **70** | Grape leaves | 3 piece | | | | | | | | | | | | |
| **71** | Orange | 1 medium | | | | | | | | | | | | |
| **72** | Watermelon | 1 we dg | | | | | | | | | | | | |
| **73** | Apricot | 1 medium | | | | | | | | | | | | |
| **74** | Coconut | 1 tbsp | | | | | | | | | | | | |

**Fifth 24hour eating recall for three day**

| **Record eating food at weekday:** | | | | |
|---|---|---|---|---|
| **Time / place** | **Food** | **Quantity / Volume** | **Frequency** | **How to prepare** |
| **Breakfast meal** | | | | |
| **Snack** | | | | |
| **Lunch meal** | | | | |
| **Snack** | | | | |
| **Dinner meal** | | | | |
| **Eating late at night** | | | | |

**Sixth: Eating Attitudes Test (EAT-26)** **Instructions**: This is a screening measure to help you determine whether you might have an eating disorder that needs professional attention. This screening measure is not designed to make a diagnosis of an eating disorder or take the place of a professional consultation. Please fill out the below form as accurately, honestly and completely as possible. There are no right or wrong answers. All of your responses are confidential.

| **Part B: Please check a response for Always Usually Often Sometimes Rarely Never each of the following statements:** | **Always** | **Usually** | **Often** | **Sometimes** | **Rarely** | **Never** |
|---|---|---|---|---|---|---|
| 1. Am terrified about being overweight. | | | | | | |
| 2. Avoid eating when I am hungry. | | | | | | |
| 3. Find myself preoccupied with food. | | | | | | |
| 4. Have gone on eating binges where I feel that I may not be able to stop. | | | | | | |
| 5. Cut my food into small pieces. | | | | | | |
| 6. Aware of the calorie content of foods that I eat. | | | | | | |
| 7. Particularly avoid food with a high carbohydrate content (i.e. bread, rice, potatoes, etc.) | | | | | | |
| 8. Feel that others would prefer if I ate more. | | | | | | |
| 9. Vomit after I have eaten. | | | | | | |
| 10. Feel extremely guilty after eating. | | | | | | |
| 11. Am preoccupied with a desire to be thinner. | | | | | | |
| 12. Think about burning up calories when I exercise. | | | | | | |
| 13. Other people think that I am too thin. | | | | | | |
| 14. Am preoccupied with the thought of having fat on my body. | | | | | | |
| 15. Take longer than others to eat my meals. | | | | | | |
| 16. Avoid foods with sugar in them. | | | | | | |
| 17. Eat diet foods. | | | | | | |

| | | | | | | |
|---|---|---|---|---|---|---|
| 18. Feel that food controls my life. | | | | | | |
| 19. Display self-control around food. | | | | | | |
| 20. Feel that others pressure me to eat. | | | | | | |
| 21. Give too much time and thought to food. | | | | | | |
| 22. Feel uncomfortable after eating sweets. | | | | | | |
| 23. Engage in dieting behavior. | | | | | | |
| 24. Like my stomach to be empty. | | | | | | |
| 25. Have the impulse to vomit after meals. | | | | | | |
| 26. Enjoy trying new rich foods. | | | | | | |

## Seventh: HBM Questionnaire

| Health Belief Model questionnaire for weight management behaviour | | | | | | |
|---|---|---|---|---|---|---|
| **Perceived severity** | | Completely disagree | Disagree | Undecided | Agree | Completely agree |
| Being overweight could … | ***Emotional/mental health subscale*** | | | | | |
| | 1. Make me feel anxious and stressed | | | | | |
| | 2. Cause others to find me less physically attractive. | | | | | |
| | 3. Make me unhappy and depressed. | | | | | |
| | 4. Lower my self-esteem. | | | | | |
| | ***Physical health/fitness subscale*** | | | | | |
| | 5. Makes it harder to do the physical activity or sports that I enjoy. | | | | | |
| | 6. Makes it harder to get enough sleep. | | | | | |
| | 7. Cause pain in my knees. | | | | | |
| | 8. Have an adverse effect on my health in years to come. | | | | | |
| | 9. increases my risk for diabetes, high blood pressure, cancer and other illnesses. | | | | | |
| | ***Social/professional subscale*** | | | | | |
| | 10. Make it harder to make friends. | | | | | |
| | 11. Makes it harder to get a job because of a lack of fitness. | | | | | |
| | 12.Take fun out of socializing with friends. | | | | | |
| | 13.Makes me unable to wear clothes I want. | | | | | |
| **Perceived susceptibility** | | Completely disagree | Disagree | Undecided | Agree | Completely agree |
| I could become susceptible to being overweightif… | ***Lifestyle subscale*** | | | | | |
| | 1. I get <30 min of moderate-intensity physical activity on most days | | | | | |
| | 2. I consume sugary beverages, foods, or snacks daily or on most days. | | | | | |
| | 3. I eat fried foods or snacks daily or on most days. | | | | | |
| | 4. I eat at fast-food restaurants ≥3 times/wk. | | | | | |
| | 5. I don’t pay attention to the amounts I eat or drink. | | | | | |
| | ***Environmental subscale*** | | | | | |
| | 6. One or both of my parents is overweight or obese. | | | | | |
| | 7. I have a genetic history of being overweight or obese. | | | | | |

| Perceived barriers | Completely disagree | Disagree | Undecided | Agree | Completely agree |
| --- | --- | --- | --- | --- | --- |
| ***Practical concerns subscale*** | | | | | |
| 1. Lower-calorie beverages, foods, and snacks are too expensive | | | | | |
| 2. Grocery shopping and preparing healthy foods would take up toomuch of my time. | | | | | |
| 3. Doing exercise/physical activity on most days would take up toomuch of my time. | | | | | |
| 4. My job/studying means more to me than adopting healthy eatingand physical activity habits | | | | | |
| ***Emotional/mental health subscale*** | | | | | |
| 5. I do not have any motivation to adopt healthyeating and physicalactivity habits | | | | | |
| 6. I enjoy eating fried foods and snacks more than baked, grilled or steamed versions | | | | | |
| 7. I enjoy consuming sugary beverages, foods, and snacks morethan lower-calorie versions | | | | | |
| 8. I often turn to food when I want to feel comforted | | | | | |
| ***Awareness subscale*** | | | | | |
| 9. I don't know where to find accurate information about achievingand maintaining a healthy weight | | | | | |
| 10.I don't know how to plan physical activity into my dailyschedule | | | | | |
| 11.I don't know where to shop for healthy beverages, foods, or snacks. | | | | | |
| 12.I don't know how to prepare low-calorie beverages, foods, or snacks. | | | | | |
| 13.I don't know how to choose low-calorie beverages, foods, orsnacks. | | | | | |

| Perceived benefits | | Completely disagree | Disagree | Undecided | Agree | Completely agree |
|---|---|---|---|---|---|---|
| It would benefit me to adopt healthy eating and physical activity habits by… | ***Emotional/mental health subscale*** | | | | | |
| | 1. Reducing depression, anxiety and stress. | | | | | |
| | 2. Helping me to improve my body image. | | | | | |
| | 3. Improve my self-esteem | | | | | |
| | 4. Improve my mood | | | | | |
| | ***Physical health/fitness subscale*** | | | | | |
| | 5. Make it easier to do the exercise/sports I enjoy | | | | | |
| | 6. Make me feel more energetic | | | | | |
| | 7. Increase my chances of having good health now and in the future | | | | | |
| | 8. Improve a symptom or health problem I have now | | | | | |
| | 9. Help me become more physically fit to improve my job performance | | | | | |
| | 10.Help me sleep better | | | | | |
| | 11.Make it easier to accomplish my daily activities | | | | | |
| | ***Social/professional subscale*** | | | | | |
| | 12.Providing a better marriage opportunity. | | | | | |
| | 13.Make me feel more comfortable around others | | | | | |
| **Cue to action** | | Completely disagree | Disagree | Undecided | Agree | Completely agree |
| I would adopt healthy eating and physical activity habits if… | ***Internal cues*** | | | | | |
| | 1. I looked in the mirror and was dissatisfied with my body | | | | | |
| | 2. My clothes fit uncomfortably tight | | | | | |
| | 3. I developed a health problem that can be improved by a healthy weight. | | | | | |
| | 4. I believe others judge me unfairly based on my weight | | | | | |
| | 5. A healthy weight would help me achieve my personal/professional goals | | | | | |
| | 6. A healthy weight would improve my depression,anxiety, or stress | | | | | |
| | ***External cues*** | | | | | |

| | | | | | | |
|---|---|---|---|---|---|---|
| | 7. A physician/nurse/dietitian advised me to be at a healthy weight | | | | | |
| | 8. A loved one developed a serious health problem from being overweight or obese | | | | | |
| | 9. A family member or close friend advised me to be at a healthy weight | | | | | |
| | 10. I was presented information about the health risks of being overweight/obese in a college course | | | | | |
| | 11. I read on a radio, television or social media website about the health risks of being overweight/obese | | | | | |
| | 12. I saw an ad for a product or service that claimed to help me be at a healthy weight | | | | | |

| **Perceived self-efficacy in dieting** | Completely disagree | Disagree | Undecided | Agree | Completely agree |
|---|---|---|---|---|---|
| ***Habits and preferences subscale*** | | | | | |
| 1. I can eat three meals regularly. | | | | | |
| 2. I can eat meals in moderate amounts. | | | | | |
| 3. I can eat fresh food rather than processed one | | | | | |
| 4. I can refrain to eat sweets like candy and cookie. | | | | | |
| 5. I can refrain to eat fatty food like fried food. | | | | | |
| 6. I can refrain to drink carbonated drink like cola | | | | | |
| 7. I can eat various foods to avoid unbalance in diet. | | | | | |
| 8. I can refrain to eat just before going to bed. | | | | | |
| 9. I can eat slowly even when hungry | | | | | |
| 10.I can stop to eat before filling stomach even the food is delicious. | | | | | |
| 11.I can get up early to eat breakfast. | | | | | |
| 12.I can refrain to watch TV or read book when I eat. | | | | | |
| 13.I can refuse to eat when my family members or friends offer foods. | | | | | |
| ***Emotional/mental health subscale*** | | | | | |
| 14.I can refrain to eat when I am bored. | | | | | |

| | | | | | |
|---|---|---|---|---|---|
| 15.I can refrain to eat when I am hungry. | | | | | |
| 16.I can refrain to eat when I am angry. | | | | | |
| 17.I can refrain to eat when I am depressed. | | | | | |
| 18.I can refrain to eat when I am anxious and excited. | | | | | |
| **Perceived self-efficacy in exercise** | Completely disagree | Disagree | Undecided | Agree | Completely agree |
| 1. I can walk for distance as far as 15 minutes' walk. | | | | | |
| 2. I can exercise until getting short of breath. | | | | | |
| 3. I can exercise in cold weather. | | | | | |
| 4. I can exercise in hot weather. | | | | | |
| 5. I can exercise with friends after university. | | | | | |
| 6. I can exercise instead of watching TV in leisure time. | | | | | |
| 7. I can use stairs instead of elevators. | | | | | |
| **Behavioral intention of weight management** | Completely disagree | Disagree | Undecided | Agree | Completely agree |
| ***Diet therapy subscale*** | | | | | |
| 1. I intend to control diet to reduce weight within six months. | | | | | |
| 2. I intend to visit dietitian to reduce weight within six months. | | | | | |
| 3. I intend to attend obese class if my college opens it. | | | | | |
| ***Exercise therapy subscale*** | | | | | |
| 4. I intend to exercise by myself regularly to reduce weight within six months. | | | | | |
| 5. I intend to attend sport centre or class to reduce weight within six months. | | | | | |

***Thank you for your cooperation in answering this questionnaire.***

# الاستبيان

**أولاً : المعلومات الاجتماعية**

| | | | |
|---|---|---|---|
| العمر: | عام | | |
| تاريخ الميلاد: | اليوم: | الشهر: | السنة: |
| المدرسة: | | | |
| الصف: | | | |
| عدد الإخوة والأخوات | | | |
| عدد أفراد العائلة الذي تعيش بالمنزل | | | |
| مستوى تعليم الأم | مرحلة المتوسط أو أقل ☐1 | مرحلة الثانوي ☐2 | مرحلة الجامعة أو أعلى ☐3 |
| مستوى تعليم الأب | مرحلة المتوسط أو أقل ☐1 | مرحلة الثانوي ☐2 | مرحلة الجامعة أو أعلى ☐3 |
| الدخل الشهري (ريال سعودي) | أقل من 5000 ☐1 | متوسط من 5000-14,999 ☐2 | أكثر أو يساوي 15,000 ☐3 |

**ثانياً : المعلومات الأنثروبومترية**

| | |
|---|---|
| **الوزن الحالي (كجم)** | |

| | |
|---|---|
| **الطول (سم)** | |
| **مؤشر كتلة الجسم**<br>**z-score** | |
| **محيط الخصر (سم)** | |
| **البدانة في منطقة البطن (سم)** | |

**ثالثاً: الأسئلة**

**أولاً: استبانة المعرفة، الممارسة، السلوك بشأن التغذية والنشاط البدني**

| الرقم | **المعرفة (1-30):** يرجى اختيار إجابة واحدة فقط تعتبرينها الأنسب لك. لا توجد أي إجابة صحيحة أو خاطئة في هذا الجزء |
|---|---|
| 1 | عدم تقبل شكل الجسم يؤثر على صحة الجسم سلباً؟<br>أ. صح<br>ب. خطأ |

| | |
|---|---|
| | ج. ال أعرف |
| 2 | تعد الخضروات والفواكه من أفضل المصادر لأللياف؟<br>أ. صح<br>ب. خطأ<br>ج. ال أعرف |
| 3 | يمكن للجميع ممارسة النشاط البدني بانتظام بغض النظر عن الحالة الصحية؟<br>أ. صح<br>ب. خطأ<br>ج. ال أعرف |
| 4 | يصنف القفز على الحبل على أنه نشاط بدني منخفض الشدة؟<br>أ. صح<br>ب. خطأ<br>ج. ال أعرف |
| 5 | االستمرار في ممارسة النشاط البدني مهم لكل األعمار؟<br>أ. صح<br>ب. خطأ<br>ج. ال أعرف |
| 6 | تعرض البطاقة الغذائية معلومات قليلة عن السعرات الحرارية؟<br>أ. صح<br>ب. خطأ<br>ج. ال أعرف |
| 7 | يحتاج المراهقون )13 - 14 سنة( إلى تناول الطعام الصحي بغض النظر عن ممارسة النشاط البدني؟<br>أ. صح<br>ب. خطأ<br>ج. ال أعرف |
| 8 | يمكن أن يقضي المراهقون )13 - 14 سنة( 4 ساعات يوميا أمام الشاشة مثل شاشة )األيباد( أو الجوال أو أي أجهزة أخرى؟<br>أ. صح<br>ب. خطأ<br>ج. ال أعرف |
| 9 | أحد األنشطة التي ال تعد سلوكا متصفا بكثرة الجلوس هو الجلوس في أثناء العزف على آالت الموسيقى؟<br>أ. صح<br>ب. خطأ<br>ج. ال أعرف |
| 10 | يمكن للمراهقين )13 - 14 سنة( عدم تناول أحد الوجبات الرئيسية يوميا؟<br>أ. صح<br>ب. خطأ<br>ج. ال أعرف |
| 11 | يصنف ركوب الدراجات على أنه من األنشطة البدنية الهوائية؟<br>أ. صح<br>ب. خطأ<br>ج. ال أعرف |

| | |
|---|---|
| 12 | مؤشر كتلة الجسم (BMI) = الوزن )كجم( / الطول )م( 2 ؟<br>أ. صح<br>ب. خطأ ج. ال<br>أعرف |
| 13 | عندما تكون كمية السعرات الحرارية المتناولة من الغذاء هي 2000 ، وكمية الطاقة المستهلكة هي 1800 سعر حراري فإن هذه الحالة تعني خسارة الوزن؟<br>أ. صح<br>ب. خطأ<br>ج. ال أعرف |
| 14 | النشاط البدني هو حركة تحدث نتيجة تقلص العضالت الكبيرة و يتطلب ذلك استهالك الطاقة؟<br>أ. صح<br>ب. خطأ<br>ج. ال أعرف |
| 15 | لمراقبة نموك تحتاج إلى قياس الوزن والطول ومؤشر كتلة الجسم؟<br>أ. صح<br>ب. خطأ<br>ج. ال أعرف |
| 16 | يمكن أن تزيد األطعمة الدهنية من احتمالية اإلصابة بالسمنة؟<br>أ. صح<br>ب. خطأ<br>ج. ال أعرف |
| 17 | تحتوي المشروبات الغازية على كميات كبيرة من األمالح المعدني؟<br>أ. صح<br>ب. خطأ<br>ج. ال أعرف |
| 18 | ال تزيد السمنة من مخاطر اإلصابة بداء السكري من النوع الثاني؟<br>أ. صح<br>ب. خطأ<br>ج. ال أعرف |
| 19 | يجب أن يكون الوقت الذي يقضى في السلوك المتصف بكثرة الجلوس 4 ساعات في اليوم؟<br>أ. صح<br>ب. خطأ<br>ج. ال أعرف |
| 20 | يفضل ممارسة نوع واحد من النشاط البدني كل يوم؟<br>أ. صح<br>ب. خطأ<br>ج. ال أعرف |
| 21 | تستطيع فاطمة أن تستبدل نمط الحياة المتصف بكثرة الجلوس باللعب بألعاب الكمبيوتر ؟<br>أ. صح<br>ب. خطأ<br>ج. ال أعرف |
| 22 | تعد األحماض الدهنية غير المشبعة آمنة إذا وجدت في الغذاء؟<br>أ. صح<br>ب. خطأ |

| | | |
|---|---|---|
| ج. لا أعرف | |
| الدردشة مع الأصدقاء في أثناء الجلوس في غرفة المعيشة لمدة ساعة أو أكثر كل يوم يمكن أن تحسن لياقتك البدنية؟<br>أ. صح<br>ب. خطأ<br>ج. لا أعرف | 23 |
| بناءً على النخلة الغذائية الصحية السعودية، تناول 2 – 3 حصص من الخضروات يوميًا سوف يجعلك تتمتعين بصحة جيدة؟<br>أ. صح<br>ب. خطأ<br>ج. لا أعرف | 24 |
| تعد تمارين الإحماء ضرورية قبل ممارسة الرياضة؟<br>أ. صح<br>ب. خطأ<br>ج. لا أعرف | 25 |
| للمشي دور حيوي في إطالة العمر؟<br>أ. صح<br>ب. خطأ<br>ج. لا أعرف | 26 |
| النشاط البدني يقلل من التوتر؟<br>أ. صح<br>ب. خطأ<br>ج. لا أعرف | 27 |
| الخمول البدني يزيد من خطر الإصابة بداء السكري من النوع الثاني؟<br>أ. صح<br>ب. خطأ<br>ج. لا أعرف | 28 |
| طلب الحجم الكبير من الطعام بسبب عروض البيع الترويجية لا يعد ممارسة غذائية صحية ؟<br>أ. صح<br>ب. خطأ<br>ج. لا أعرف | 29 |
| شرب الماء قبل، في أثناء، وبعد المشي مهم لصحة جسمك؟<br>أ. صح<br>ب. خطأ<br>ج. لا أعرف | 30 |

**المواقف )1-22(:** يرجى اختيار إجابة واحدة فقط تعبيرًا عما ألانسب لك. لا توجد أي إجابة صحيحة أو خاطئة في هذا الجزء

| أوافق بشدة | أوافق | أحيانًا | لا أوافق | لا أوافق بشدة | الجملة | |
|---|---|---|---|---|---|---|
| | | | | | أعتقد أن تناول الطعام الصحي أكثر أهمية من | 1 |

| | | | | | | |
|---|---|---|---|---|---|---|
| | ممارسة النشاط البدني لصحة أجسامنا | | | | | |
| 2 | طريقة الطهي لن تؤثر في اختياري للطعام عندما أكون خارج المنزل | | | | | |
| 3 | أعتقد أن عدم تناول وجبة اإلفطار أمر غير جيد لصحة جسمي | | | | | |
| 4 | أعتقد أن تناول الطعام الصحي غير مهم لصحة الجسم خالل فترة النمو | | | | | |
| 5 | أعتقد أنه يمكنني زيادة نشاطي البدني تدريجيا ألحصول على الفوائد الصحية | | | | | |
| 6 | أعتقد أن مساعدة أسرتي في األعمال المنزلية مهم لزيادة النشاط البدني | | | | | |
| 7 | أحب أن أستخدم الجوال ألكثر من ثالث ساعات كل يوم | | | | | |
| 8 | ممارسة النشاط البدني كالمشي كل يوم على أن ال تزيد المدة عن 10 دقيقة | | | | | |
| 9 | أعتقد أن السمنة ال ترتبط بتناول كمية كبيرة من السعرات الحرارية | | | | | |
| 10 | أعتقد أن ممارسة النشاط البدني يجب أن يكون خارج المنزل | | | | | |
| 11 | [illegible] أن أتبع النخلة الغذائية الصحية؛ ألنها تساعدني على تناول الطعام الصحي | | | | | |
| 12 | أعتقد أن هناك عالقة بين الغذاء الصحي والتحصيل الدراسي | | | | | |
| 13 | أعتقد أن السلوك المتصف بكثرة الجلوس ال يؤثر على وزن الجسم | | | | | |
| 14 | [illegible] أن صحة الجسم تتأثر بعدم التوازن بين كمية الطاقة الغذائية المتناولة وكمية استهالك الطاقة المستهلكة | | | | | |
| 15 | أعتقد أن ما آكله كل يوم لن يؤثر في صحة جسدي في المستقبل | | | | | |
| 16 | يجب أن يوصى بتناول األطعمة النشوية في كل وجبة رئيسية | | | | | |
| 17 | أعتقد أن توصيات النشاط البدني تساعدني في أن ألكون طالبا نشطا | | | | | |
| 18 | أعتقد أنه يمكنني القيام بنشاط بدني معتدل الشدة | | | | | |

| | | | | | | |
|---|---|---|---|---|---|---|
| | لمدة لا تقل عن 60 دقيقة يوميًا | | | | | |
| 19 | تصوري لشكل جسمي لا يمكن أن يؤثر على حالتي الصحية | | | | | |
| 20 | أعتقد أن ممارسة النشاط البدني بكثرة يمكن أن يساعدني على تجنب داء السكري من النوع الثاني | | | | | |
| 21 | كمية السعرات الحرارية المكتوبة على البطاقة الغذائية على اختياري للطعام | | | | | |
| 22 | أعتقد أن ممارسة النشاط البدني يمكن أن يساعدني في التحكم في وزن جسمي | | | | | |

**الممارسة (1-21):** يرجى اختيار إجابة واحدة فقط تعتبرينها الأنسب لك. لا توجد أي إجابة صحيحة أو خاطئة في هذا الجزء

| | **كم مرة** | **غالبًا جدا** | **غالبًا** | **أحيانًا** | **نادرًا** | **أبدًا** |
|---|---|---|---|---|---|---|
| **1** | تمارسين أي نوع من أنواع النشاط البدني في المكان والزمان المناسب | | | | | |
| **2** | لا تشتين بهدف ممارسة النشاط البدني بدون عائلتك أو صديقاتك | | | | | |
| **3** | تقضين 4 ساعات أو أكثر في السلوك المتصف بكثرة الجلوس مثل مشاهدة التلفاز أو الدردشة مع الأصدقاء في كل اليوم | | | | | |
| **4** | تطلبين الطعام المقلي بدلا من الطعام المشوي | | | | | |
| **5** | ربين الماء عند ممارسة النشاط البدني المعتدل الشدة لمدة 60 دقيقة أو أكثر | | | | | |
| **6** | لا تقرئين القيمة الغذائية الموجودة على البطاقة الغذائية عند شراء الطعام | | | | | |
| **7** | بدلين الوقت الذي تقضينه أمام الشاشة مثل شاشة الجوال أو (الآيباد) بالنشاط البدني | | | | | |
| **8** | تتجنبين أوقات الوجبات الرئيسية من أجل إنقاص وزنك | | | | | |
| **9** | تقومين بزيادة النشاط البدني كلما سمحت لك الفرصة كمساعدة والدتك في الأعمال المنزلية | | | | | |
| **10** | تمارسين النشاط البدني المعتدل الشدة كل يوم لمدة 60 دقيقة | | | | | |
| **11** | لا تقومين بالنشاط البدني اليومي | | | | | |

| | | | | | | |
|---|---|---|---|---|---|---|
| | | | | | تضمنين خضروات وفواكه متنوعة في وجباتك اليومية | 12 |
| | | | | | تمشين بهدف ممارسة النشاط البدني يوميا | 13 |
| | | | | | تتجنبين تناول الأطعمة الغنية بالدهون عندما تتناولين الطعام خارج المنزل لتتجنبي السمنة | 14 |
| | | | | | تنظرين إلى المرآة لتتحققي من شكل جسمك | 15 |
| | | | | | تطلبين المشروبات الغازية عندما تتناولين الطعام خارج المنزل | 16 |
| | | | | | تمارسين النشاط البدني لتحافظي على صحة جسمك | 17 |
| | | | | | تقومين بتمرينات الإحماء قبل الرياضة | 18 |
| | | | | | تقيسين وزنك من أجل الحفاظ أو الوصول إلى الوزن الطبيعي | 19 |
| | | | | | تستبدلين النشاط البدني المنخفض الشدة بالنشاط البدني المعتدل الشدة | 20 |
| | | | | | تتجنبين شرب الحليب أو منتجات الألبان في طبقك كل يوم | 21 |

**ثانيا ً: استبيان النشاط البدني**

نحن نحاول معرفة مستوى نشاطك البدني من آخر 7 أيام (في الأسبوع الأخير). وهذا يشمل الرياضة أو الرقص الذي يجعلك تعرق أو تجعل ساقيك يشعران بالتعب ، أو الألعاب التي تجعلك تتنفس بصعوبة ، مثل العلامات ، والقفز ، والجري ، والتسلق ، وغيرها.

**تذكر:**

أ. لا توجد إجابات صحيحة أو خاطئة - هذا ليس اختبارا ً.

ب. . يرجى الإجابة على جميع الأسئلة بصدق ودقة بقدر ما تستطيع - وهذا مهم جدا.

| **الأسئلة** | **عدد المرات** | | | | |
|---|---|---|---|---|---|
| **النشاط البدني في وقت فراغك: هل قمت بأي من الأنشطة التالية في الـ 7 أيام الماضية (الأسبوع الماضي)؟ إذا كان الجواب نعم، كم مرة؟ (حدد اختيار واحد فقط).** | **لا** | **1-2 مرات** | **3-4 مرات** | **5-6 مرات** | **أكثر من 7 مرات** |
| أ. نط الحبل | | | | | |
| ب. العلامة | | | | | |
| ج. لعبة الكراسي | | | | | |
| د. الرقص بالطرق | | | | | |
| ه. سحب الحبل | | | | | |
| و. التزلج بأحذية التزلج | | | | | |
| ز. المشي لممارسة الرياضة | | | | | |
| ح. الغميضة | | | | | |
| ط. الركض أو الجري | | | | | |
| ي. تمارين لياقة بدنية | | | | | |
| ك. السباحة | | | | | |
| ل. الرقص | | | | | |
| م. كرة القدم | | | | | |
| ن. كرة السلة | | | | | |
| س. الكرة الطائرة | | | | | |
| ع. الدراجة الهوائية | | | | | |
| ف. أخرى:........................................... | | | | | |

| | | | | | |
|---|---|---|---|---|---|
| | | | | | ص. أخرى:........................................... |

**2 خالل األيام السبعة الماضية ، خالل فصول النشاط البدنية ، كم مرة مارست فيه نشاطا بدنيا مرتفع الشدة )اللعب بقوة ،**

**الجري ، القفز ، الرمي(؟ )تحقق من واحد فقط.(**

أ. ال أقوم بنشاط رياضي
ب. بصعوبة جدا
ج. بعض األحيان
د. غالبا ج
ه. دائما

**3. خالل األيام السبعة الماضية ، ماذا فعلت في معظم الوقت الراحة ؟ )تحقق من واحد فقط(:**

أ. جلست )أتحدث، أقرأ ، أقوم بواجباتي المدرسية(
ب. وقفت أو مشيت حول المكان
ج. ركضت أو لعبت قليال جدا
د. ركضت أو لعبت قليال
ه. ركضت أو لعبت بقوة في معظم الوقت

**4. خالل األيام السبعة الماضية ، ماذا كنت تفعل عادة عند الغداء )إلى جانب تناول الغداء(؟ )تحقق من واحد فقط(.**

أ. جلست )أتحدث، أقرأ ، أقوم بواجباتي المدرسية(
ب. وقفت أو مشيت حول المكان
ج. ركضت أو لعبت قليال جدا
د. ركضت أو لعبت قلي ال
ه. ركضت أو لعبت بقوة في معظم الوقت

**5- خالل األيام السبعة الماضية ، كم يوم قمت فيه بممارسة الرياضة أو الرقص أو اللعبو كنت فيها نشيطا للغاية بعد المدرسة مباشرة )تحقق من واحد فقط(.**

أ. ال شيء
ب. مرة واحدة في األسبوع الماضي
ج. 2 أو 3 مرات في األسبوع الماضي
د. 4 مرات في األسبوع الماضي
ه. هـ. 5 مرات في األسبوع الماضي

**6- خالل األيام السبعة الماضية ، كم عدد المرات التي قمت فيها بالرياضة ، أو الرقص ، أو ممارسة األلعاب التي كنت**

| |
|---|
| فيها نشيطًا للغاية في فترة المساء؟ (تحقق من واحد فقط.)<br><br>أ. الشيء<br>ب. مرة واحدة في الأسبوع الماضي<br>ج. أو 3 مرات في الأسبوع الماضي<br>د. أو 5 الأسبوع الماضي<br>ه. 6 أو 7 مرات في الأسبوع الماضي |
| **7- خلال عطلة نهاية الأسبوع الماضي ، كم مرة قمت فيها بممارسة الرياضة أو الرقص أو ممارسة الألعاب و كنت نشيطًا للغاية؟ (تحقق من واحد فقط.)**<br><br>أ. أ.الشيء<br>ب. 1 مرة<br>ت. 2 - 3 مرات<br>ث. 4 - 5 مرات ج.<br>6 مرات أو أكثر |
| **8. أي مما يلي يصف لك أفضل شيء خلال الأيام السبعة الماضية ؟ اقرأ جميع العبارات الخمس قبل أن تقرر الإجابة الوحيدة التي تصفك**<br><br>أ. لقد قضيت كل أو معظم وقت فراغي في القيام بأنشطة تشمل جهد بدني قليل.<br>ب. أحيانًا (1-2 مرات في الأسبوع الماضي) قمت بأنشطة بدنية في وقت الفراغ (على سبيل المثال: لعبت الرياضة ، جريت ، سبحت ، ركبت الدراجة ، تمارين اللياقة البدنية). ج.<br>غالبًا (3 - 4 مرات في الأسبوع الماضي) أقوم بأنشطة بدنية في وقت الفراغ<br>. في كثير من الأحيان (5 - 6 مرات في الأسبوع الماضي) أقوم بأنشطة بدنية في وقت الفراغ<br>ه غالبًا جدًا (7 مرات أو أكثر في الأسبوع الماضي) أقوم بأنشطة بدنية في وقت الفراغ |

**9. حدد عدد المرات التي مارست فيها النشاط البدني مثال ) ممارسة الرياضة ، الألعاب ، الرقص ، أو أي نشاط بدني آخر( خلال أيام الأسبوع الماضي.**

| أيام الأسبوع | لا | قليلا | متوسط | غالبا | غالبا جدا |
|---|---|---|---|---|---|
| **الأحد** | | | | | |
| **الاثنين** | | | | | |
| **الثلاثاء** | | | | | |
| **الأربعاء** | | | | | |
| **الخميس** | | | | | |
| **الجمعة** | | | | | |
| **السبت** | | | | | |

**10. هل كنت مريضا في الأسبوع الماضي ، أو منعك شيء ما من القيام بأنشطتك البدنية الطبيعية؟ )ضع علامة على واحدة.(**

○ نعم

○ لا

إذا كان الجواب نعم ، ما الذي منعك؟

..........................................................................................................................

..........................................................................................................................

**ثالثا : الأنشطة المستقرة**

**الآن ، بعض الأسئلة حول الأنشطة المتصفة بكثرة الجلوس**

عموما ، لديك حوالي 8 ساعات من وقت الفراغ قبل وبعد المدرسة ، وبعضها يقضى في الجلوس.يمكنك القيام بأنشطة متعددة في نفس الوقت )اللعب بالهواتف الذكية أمام التلفزيون(

لذا يرجى تقدير مقدار الوقت الذي تم إنفاقه على كل نشاط من خلال إجمالي الوقت المستغرق في القيام بالأنشطة تكرر في أسبوع مدرسي عادي ، واكتب المدة التي تقضيها في القيام بالأنشطة التالية قبل وبعد المدرسة كل يوم ، اترك المكان فارغا إذا لم تقم بهذا النشاط.

| **الأنشطة / المدة الزمنية** | **الأحد** | | | **الاثنين** | | | **الثلاثاء** | | | **الأربعاء** | | | **الخميس** | | |
|---|---|---|---|---|---|---|---|---|---|---|---|---|---|---|---|
| | دقائق | | ساعة | دقائق | | ساعة | دقائق | | ساعة | دقائق | | ساعة | دقائق | | ساعة |
| مشاهدة التلفزيون | | | | | | | | | | | | | | | |
| مشاهدة أشرطة الفيديو / أقراص الفيديو الرقمية (دي في دي) | | | | | | | | | | | | | | | |
| استخدام الكمبيوتر للمتعة | | | | | | | | | | | | | | | |
| اللعب على الهاتف الذكي أو الآيباد | | | | | | | | | | | | | | | |
| لعب ألعاب الكمبيوتر أو الفيديو (نانتندو، إكس بوكس، بلايستيشن، وي) | | | | | | | | | | | | | | | |
| استخدام الكمبيوتر في أداء الواجب المنزلي | | | | | | | | | | | | | | | |
| أداء الواجبات المنزلية بدون استخدام الكمبيوتر | | | | | | | | | | | | | | | |
| القراءة للمتعة | | | | | | | | | | | | | | | |
| الدراسة | | | | | | | | | | | | | | | |
| السفر (سيارة / حافلة / قطار) | | | | | | | | | | | | | | | |
| ممارسة الحرف أو الهوايات | | | | | | | | | | | | | | | |

| | | | | | | | | | | | | | | | |
|---|---|---|---|---|---|---|---|---|---|---|---|---|---|---|---|
| الدردشة مع الأصدقاء / على الهاتف / الاسترخاء | | | | | | | | | | | | | | | |
| لعب / العزف على آلة موسيقية | | | | | | | | | | | | | | | |

عموما، لديك حوالي 16 ساعات من وقت الفراغ قبل وبعد المدرسة، وبعضها يقضى في الجلوس. يمكنك القيام بأنشطة متعددة في نفس الوقت (اللعب بالهواتف الذكية أمام التلفزيون)

لذا يرجى تقدير مقدار الوقت الذي تم إنفاقه على كل نشاط من خلال إجمالي الوقت المستغرق في القيام بالأنشطة. فكر في أسبوع مدرسي عادي، واكتب المدة التي تقضيها في القيام بالأنشطة التالية قبل وبعد المدرسة كل يوم، اترك المكان فارغا إذا لم تقم بهذا النشاط.

| الأنشطة / المدة الزمنية | الجمعة | | | السبت | | |
|---|---|---|---|---|---|---|
| | دقائق | | ساعة | دقائق | | ساعة |
| مشاهدة التلفزيون | | | | | | |
| مشاهدة أشرطة الفيديو / أقراص الفيديو الرقمية (دي في دي) | | | | | | |
| استخدام الكمبيوتر للمتعة | | | | | | |
| اللعب على الهاتف الذكي أو الآيباد | | | | | | |
| لعب ألعاب الكمبيوتر أو الفيديو (نينتندو، إكس بوكس، بلايستيشن، وي) | | | | | | |
| استخدام الكمبيوتر في أداء الواجب المنزلي | | | | | | |
| أداء الواجبات المنزلية بدون استخدام الكمبيوتر | | | | | | |
| القراءة للمتعة | | | | | | |

| | | | | | | |
|---|---|---|---|---|---|---|
| الدراسة | | | | | | |
| السفر )سيارة / حافلة / قطار( | | | | | | |
| ممارسة الحرف أو الهوايات | | | | | | |
| وضع الدردشة مع األصدقاء / على الهاتف / االسترخاء | | | | | | |
| لعب / العزف على آلة موسيقية | | | | | | |
| الذهاب إلى المسجد ألداء الصالة | | | | | | |

**رابعا : استبيان تكرار تناول الغذاء لألطفال و المراهقين السعوديين، إذا سمحت أجب عن مدى استهالكك للطعام خالل الفترة الماضية**

| الرقم | اسم الطعام | الحجم الطبيعي | غير الحجم الطبيعي | | / يوم | | | | / أسبوع | | | / شهر | | |
|---|---|---|---|---|---|---|---|---|---|---|---|---|---|---|
| **الحبوب** | | | **أصغر** | **أكبر** | **1 مرة** | **3-2 مرة** | **5-4 مرة** | **7-6 مرة** | **2-1 مرة** | **4-3 مرة** | **6-5 مرة** | **1 مرة** | **3-2 مرة** | **وال مرة** |
| **1** | خبز | 1 شريحة | | | | | | | | | | | | |
| **2** | معكرونة | 1 كوب/250 مل | | | | | | | | | | | | |
| **3** | كورن فلكس | 1 كوب/250 مل | | | | | | | | | | | | |
| **4** | نوشار | 1 كوب/250 مل | | | | | | | | | | | | |
| **5** | حمص | 2 ملعقة كبيرة | | | | | | | | | | | | |
| **6** | فالفل | 2 قطع متوسط | | | | | | | | | | | | |
| **7** | بسكويت | 1 بسكويت | | | | | | | | | | | | |
| **اللحوم واألسماك و الدواجن** | | | **أصغر** | **أكبر** | **1 مرة** | **3-2 مرة** | **5 -4 مرة** | **7-6 مرة** | **2-1 مرة** | **4-3 مرة** | **6-5 مرة** | **1 مرة** | **3-2 مرة** | **وال مرة** |
| **8** | بيض | 1 بيضة | | | | | | | | | | | | |
| **9** | سجق | 85 غرام | | | | | | | | | | | | |

| | | | | | | | | | | | | | | |
|---|---|---|---|---|---|---|---|---|---|---|---|---|---|---|
| 10 | سمك مقلي | 170 غرام | | | | | | | | | | | | |
| 11 | مورتديلا | 85 غرام | | | | | | | | | | | | |
| 12 | تونة بالزيت | 1 علبة | | | | | | | | | | | | |
| 13 | دجاج مقلي | 85 غرام | | | | | | | | | | | | |
| 14 | دجاج مسلوق | 85 غرام | | | | | | | | | | | | |
| 15 | دجاجمشوي | 85 غرام | | | | | | | | | | | | |
| 16 | حساء دجاج | 1 كوب 250 مل | | | | | | | | | | | | |
| 17 | لحم غنم مسلوق | 85 غرام | | | | | | | | | | | | |
| 18 | ـنة كباب لحم | 85 غرام | | | | | | | | | | | | |
| **الأطباق المختلطة** | | | **أصغر** | **أكبر** | **1 مرة** | **3-2 مرة** | **5-4 مرة** | **7-6 مرة** | **2-1 مرة** | **4-3 مرة** | **6-5 مرة** | **1 مرة** | **3-2 مرة** | **وال مرة** |
| 19 | جر الدجاج بالجبن | 1 نطيرة متوسطة | | | | | | | | | | | | |
| 20 | مطبق | 85 غرام | | | | | | | | | | | | |
| 21 | أرز كبسة | ½ كوب / 125 مل | | | | | | | | | | | | |
| 22 | اندويتش جبن | 1 نطيرة متوسطة | | | | | | | | | | | | |
| 23 | ساندويتش بيض | 1 نطيرة متوسطة | | | | | | | | | | | | |
| 24 | بيتزا | 1 شريحة متوسط | | | | | | | | | | | | |
| 25 | اندويتش لحم | 1 نطيرة متوسطة | | | | | | | | | | | | |
| 26 | سمبوسة لحم | 1 حبة | | | | | | | | | | | | |
| 27 | رجر اللحم بالجبن | 1 نطيرة متوسطة | | | | | | | | | | | | |
| 28 | اندويتش كبدة | 1 نطيرة متوسطة | | | | | | | | | | | | |
| 29 | شاورما الدجاج | 1 نطيرة متوسطة | | | | | | | | | | | | |
| 30 | ساندويش مشكل | 1 نطيرة متوسطة | | | | | | | | | | | | |
| **منتجات الألبان** | | | **أصغر** | **أكبر** | **1 مرة** | **3-2 مرة** | **5-4 مرة** | **7-6 مرة** | **2-1 مرة** | **4-3 مرة** | **6-5 مرة** | **1 مرة** | **3-2 مرة** | **وال مرة** |

| | | | | | | | | | | | | | | |
|---|---|---|---|---|---|---|---|---|---|---|---|---|---|---|
| **31** | جبن كريم | 2 ملعقة كبيرة /30 مل | | | | | | | | | | | | |
| **32** | بادي كامل الدهون | 170 غرام | | | | | | | | | | | | |
| **33** | حليب كامل الدهون | 1 كوب 250 مل | | | | | | | | | | | | |
| **34** | حليب قليل الدهون | 1 كوب 250 مل | | | | | | | | | | | | |
| **35** | أيس كريم | ½ كوب 125 مل | | | | | | | | | | | | |
| **36** | ملك شيك | 1 كوب 250 مل | | | | | | | | | | | | |
| **المشروبات** | | | **أصغر** | **أكبر** | **1 مرة** | **3-2 مرة** | **5 -4 مرة** | **7-6 مرة** | **2-1 مرة** | **4-3 مرة** | **6-5 مرة** | **1 مرة** | **3-2 مرة** | **وال مرة** |
| **37** | شراب البرتقال | ½ كوب175 مل | | | | | | | | | | | | |
| **38** | عصير معلب غير محلى | ½ كوب175 مل | | | | | | | | | | | | |
| **39** | محضر مع الحليب | 1 كوب250 مل | | | | | | | | | | | | |
| **40** | ير برتقال طازج | ½ كوب175 مل | | | | | | | | | | | | |
| **41** | مشروبات غازية | 1 كوب250 مل | | | | | | | | | | | | |
| **42** | عصير تفاح | ½ كوب175 مل | | | | | | | | | | | | |
| **43** | كابتشينو | 1 كوب250 مل | | | | | | | | | | | | |
| **44** | قهوة سريعة التحضير | 1 كوب250 مل | | | | | | | | | | | | |
| **45** | عصير عنب | ½ كوب175 مل | | | | | | | | | | | | |
| **الحلويات والمخبوزات** | | | **أصغر** | **أكبر** | **1 مرة** | **3-2 مرة** | **5 -4 مرة** | **7 -6 مرة** | **2-1 مرة** | **4-3 مرة** | **6-5 مرة** | **1 مرة** | **3-2 مرة** | **وال مرة** |
| **46** | معجنات ( نطاير) | 1 نطيرة متوسطة | | | | | | | | | | | | |
| **47** | عسل | 1 ملعقة كبيرة | | | | | | | | | | | | |
| **48** | حلويات مشكلة | ½ كوب | | | | | | | | | | | | |
| **49** | سكر | 1 ملعقة كبيرة | | | | | | | | | | | | |
| **50** | بسبوسة | 1 شريحة | | | | | | | | | | | | |

| | | | | | | | | | | | | | | |
|---|---|---|---|---|---|---|---|---|---|---|---|---|---|---|
| 51 | كرواسون | 1 فطيرة متوسطة | | | | | | | | | | | | |
| 52 | معمول | 1 قطعة | | | | | | | | | | | | |
| 53 | بقلاوة | 1 قطعة | | | | | | | | | | | | |
| 54 | كعك | 1 قطعة متوسطة | | | | | | | | | | | | |
| 55 | دونات | 1 قطعة متوسطة | | | | | | | | | | | | |
| 56 | كريمة الحلويات | ½ كوب | | | | | | | | | | | | |
| 57 | كنافة بالقشطة | ½ كوب | | | | | | | | | | | | |
| 58 | لقمة القاضي | 1 قطعة متوسطة | | | | | | | | | | | | |
| **الفواكه والخضروات** | | | **أصغر** | **أكبر** | **1 مرة** | **3-2 مرة** | **4- 5 مرة** | **6- 7 مرة** | **2-1 مرة** | **4-3 مرة** | **6-5 مرة** | **1 مرة** | **3-2 مرة** | **وال مرة** |
| 59 | تمر | 1 حبة متوسطة | | | | | | | | | | | | |
| 60 | حساء الخضروات | 1 كوب 250 مل | | | | | | | | | | | | |
| 61 | خضروات مطبوخة | ½ كوب175 مل | | | | | | | | | | | | |
| 62 | فول مدمس | ½ كوب175 مل | | | | | | | | | | | | |
| 63 | مكسرات منوعة | ½ كوب | | | | | | | | | | | | |
| 64 | رقائق البطاطس | 28 غرام علبة | | | | | | | | | | | | |
| 65 | بطاطس مسلوقة | 1 حبة متوسطة | | | | | | | | | | | | |
| 66 | زعتر | 1 ملعقة كبيرة | | | | | | | | | | | | |
| 67 | زيتون أسود | 3 قطع | | | | | | | | | | | | |
| 68 | سلطة خضار | ½ كوب | | | | | | | | | | | | |
| 69 | طماطم طازجة | 1 حبة متوسطة | | | | | | | | | | | | |
| 70 | ورق عنب | 3 قطع | | | | | | | | | | | | |
| 71 | برتقال | 1 حبة متوسطة | | | | | | | | | | | | |
| 72 | بطيخ | 1 شريحة | | | | | | | | | | | | |
| 73 | مشمش | 1 حبة متوسطة | | | | | | | | | | | | |
| 74 | جوز الهند | 1 ملعقة كبيرة | | | | | | | | | | | | |

**خامسا ً : سجل المتناول من األطعمة خالل 24 ساعة خالل ثالثة أيام**

| سجل تناول الطعام في أيام األسبوع: | | | | |
|---|---|---|---|---|
| **المكان / الوقت** | **طعام** | **الكمية / حجم** | **تكرر** | **طريقة التحضير** |
| **وجبة اإلفطار** | | | | |

| | | | | |
|---|---|---|---|---|
| | | | | وجبة خفيفة |
| | | | | وجبة الغداء |
| | | | | وجبة خفيفة |
| | | | | وجبة العشاء |
| | | | | تناول الطعام في وقت متأخر من الليل |

**سادساً : استبانه المواقف الغذائية عند الطالب**

ذه االستبانه إلى معرفة المواقف الغذائية ، يرجى التعاون معنا بتزويدنا بالمعلومات المطلوبة ، علماً بأن هذه المعلومات سرية ولن تستعمل ألي أغراض أخرى خارجة عن نطاق الدراسة

| العبارات | دائماً | عادة )سلوك مألوف كثير التكرار( | غالباً )يتكرر السلوك في أغلب األحيان( | أحياناً )يتكرر السلوك مرات قليلة( | نادراً | مطلقاً )أبداً ( |
|---|---|---|---|---|---|---|
| 1.إنني قلق من أن يكون وزني زائداً | | | | | | |
| 2.أتجنب األكل عندما أكون جائعاً | | | | | | |
| أجد نفسي مشغول بالطعام/ باألكل | | | | | | |
| 4.أتناول الطعام بشراهة ) كمية كبيرة من الطعام(، بحيث اشعر أنه ال يمكنني التوقف | | | | | | |
| 5.أقطع طعامي لقطع صغيرة | | | | | | |
| 6.أعرف محتوى السعرات الحرارية لألغذية التي أتناولها | | | | | | |
| 7.أتجنب بشكل خاص األطعمة التي تحتوي على كمية عالية من ربوهيدرات كالخبز ، و األرز ، والبطاطا | | | | | | |
| 8.أشعر بأن االخرين يفضلون لو أنني أكلت أكثر | | | | | | |

| | | | | | | |
|---|---|---|---|---|---|---|
| 9.أتقيأ إراديا (أحاول التقيؤ) بعد تناول الطعام | | | | | | |
| 10.أشعر بذنب كبير بعد الأكل | | | | | | |
| 11.أتذكر أن أكون نحيفا | | | | | | |
| 12.أفكر في حرق رات الحرارية عند التمرين الرياضي | | | | | | |
| 13.يعتقد الأشخاص الآخرين بأني نحيف جدا | | | | | | |
| 1.أنا منشغل بفكرة أن يوجد دهن متراكم في جسمي | | | | | | |
| **العبارات** | **دائما** | **عادة (سلوك مألوف كثير التكرار)** | **غالبا (يتكرر السلوك في أغلب الأحيان)** | **أحيانا (يتكرر السلوك مرات قليلة)** | **نادرا** | **مطلقا (أبدا)** |
| 15.أستغرق وقتا أطول من الآخرين في تناول الطعام | | | | | | |
| 16.أتجنب الأطعمة المحتوية على السكر | | | | | | |
| 17.أتناول الأغذية المخصصة للرجيم | | | | | | |
| 18.أشعر الطعام يتحكم بحياتي وسلوكي | | | | | | |
| 19.أستطيع أن أتحكم بكمية الطعام الذي أتناوله | | | | | | |
| 20.أشعر بأن الآخرين يضغطون علي لأتناول الطعام | | | | | | |
| 21.أعطي الكثير من الوقت و التفكير للطعام (أن الطعام يشغل تفكيري) | | | | | | |

| | | | | | | |
|---|---|---|---|---|---|---|
| 2.أشعر بعدم الارتياح بعد تناول الحلويات | | | | | | |
| 23.اتبع نظام رجيم غذائي)حمية معينة( | | | | | | |
| .أحب أن تكون معدتي فارغة من الطعام | | | | | | |
| 25.أشعر أنه لدي اندفاع للتقيؤ بعد تناول الوجبات )أحاول التقيؤ( | | | | | | |
| 2.استمتع بتناول أغذية جديدة غنية بالسعرات الحرارية | | | | | | |
| **المجموع النهائي** | | | | | | |

**سابعا : استبيان نموذج المعتقدات الصحية**

| **استبيان نموذج المعتقدات الصحية لسلوك إدارة الوزن** | | | | | | |
|---|---|---|---|---|---|---|
| **الخطورة المتصورة** | | **لا أوافق مطلقا** | **لا أوافق** | **غير متأكد** | **أوافق** | **موافق تماما** |
| **زيادة الوزن يمكن ان ...** | **المقياس الفرعي :الصحة العاطفية /الصحة العقلية** | | | | | |
| | 1. تجعلني أشعر بالقلق والتوتر | | | | | |
| | 2. تجعل الآخرين يجدونني أقل جاذبية جسديا. | | | | | |
| | 3. تجعلني غير سعيد ومكتئب. | | | | | |
| | 4. تقلل من تقديري لذاتي. | | | | | |
| | **المقياس الفرعي :الصحة البدنية / اللياقة البدنية** | | | | | |

| | | | | | | |
|---|---|---|---|---|---|---|
| | | | | | 5. تجعل من الصعب القيام بالنشاط البدني أو الرياضة التي أستمتع بها. | |
| | | | | | 6. تجعل من الصعب الحصول على قسط كاف من النوم. | |
| | | | | | 7. تسبب آلام في ركبتي. | |
| | | | | | 8. يكون لها تأثير سلبي على صحتي في السنوات القادمة. | |
| | | | | | 9. تزيد من مخاطر االصابة بمرض السكر وارتفاع ضغط الدم والسرطان وأمراض أخرى. | |
| | | | | | **المقياس الفرعي: االجتماعي / المهني** | |
| | | | | | 10. تجعل من الصعب تكوين صداقات. | |
| | | | | | 11. يجعل من الصعب الحصول على عمل بسبب نقص اللياقة. | |
| | | | | | 12. تأخذ المرح من التواصل مع األصدقاء | |
| | | | | | 13. تجعلني غير قادر على ارتداء المالبس التي أريدها. | |
| | | | | | | |
| **موافق تماما** | **أوافق** | **غير متأكد** | **ال أوافق** | **ال أوافق مطلقا** | **القابلية المتصورة** | |

| | | | | | | |
|---|---|---|---|---|---|---|
| يمكن أن أصبح عرضة لزيادة الوزن إذا | المقياس الفرعي :نمط الحياة | | | | | |
| | ت على أقل من 30 دقيقة من النشاط البدني المتوسط الشدة في معظم الأيام | | | | | |
| | 2. أستهلكت المشروبات أو الأطعمةأو الوجبات الخفيفة السكرية يومي ا أو في معظم الأيام | | | | | |
| | 3. تناولت الأطعمة المقلية أو الوجبات الخفيفة يومي ا أو في معظم الأيام. | | | | | |
| | أنا أكلت في مطاعم الوجبات السريعة ≥3 مرات / أسبوع | | | | | |
| | 5. أنا لم ألاحظ الكميات التي أتناولها أو أشربها | | | | | |
| المقياس الفرعي : البيئة | | | | | | |
| | 6. يعاني أحد والد ي أو كليهما من زيادة الوزن أو السمنة. | | | | | |
| | 7. لدي تاريخ وراثي من زيادة الوزن أو السمنة. | | | | | |
| | العوائق المتصوره | لا أوافق مطلقا | لا أوافق | غير متأكد | أوافق | موافق تماما |
| المقياس الفرعي : مخاوف عملية | | | | | | |
| | المشروبات والأطعمة والوجبات الخفيفة منخفضة السعرات باهظة الثمن | | | | | |
| | 2. يستغرق شراء البقالة وتحضير الأطعمة الصحية الكثير من وقتي. | | | | | |
| | 3. ممارسة الرياضة / النشاط البدني | | | | | |

| | | | | | | |
|---|---|---|---|---|---|---|
| | في معظم الأيام سيستغرق الكثير من وقتي. | | | | | |
| | وظيفتي / دراستي تعني لي أكثر من تبني عادات الأكل الصحي والنشاط البدني | | | | | |
| **المقياس الفرعي : الصحة العاطفية / العقلية** | | | | | | |
| | ليس لدي أي دافع لتبني عادات الأكل الصحي والنشاط البدني | | | | | |
| | 6. أنا أستمتع بتناول الأطعمة المقلية والوجبات الخفيفة أكثر من المخبوزات أو المشوية أوعلى البخار | | | | | |
| | 7. أستمتع بتناول المشروبات والأطعمة والوجبات الخفيفة السكرية أكثر من الأنواع منخفضة السعرات الحرارية | | | | | |
| | 8. غالبـــًا ما ألجأ إلى الطعام عندما أريد أن أشعر بالراحة | | | | | |
| **المقياس الفرعي : التوعية** | | | | | | |
| | 9. لا أعرف من أين أجد معلومات دقيقة حول الوصول إلى وزن صحي والحفاظ عليه | | | | | |
| | 1. لا أعرف كيف أخطط للنشاط البدني في جدول أعمالي اليومي | | | | | |
| | 11. لا أعرف من أين أتسوق للحصول على مشروبات أو أطعمة صحية أو وجبات خفيفة. | | | | | |
| | 12. لا أعرف كيفية تحضير المشروبات أو الأطعمة منخفضة | | | | | |

| | | | | | | |
|---|---|---|---|---|---|---|
| | السعرات الحرارية أو وجبات خفيفة. | | | | | |
| | ل أعرف كيف أختار المشروبات أو الأطعمة أو الوجبات الخفيفة منخفضة السعرات الحرارية. | | | | | |
| | **الفائدة المتصورة** | **ال أوافق مطلقا** | **ال أوافق** | **غير متأكد** | **أوافق** | **موافق تماما** |
| **سوف يفيدني تبني عادات األكل الصحي والنشاط البدني من خالل** | **المقياس الفرعي :الصحة العاطفية / العقلية** | | | | | |
| | 1- تزيل االكتئاب والقلق والتوتر. | | | | | |
| | 2- مساعدتي في تحسين صورة جسدي | | | | | |
| | 3- تحسين تقديري لذاتي | | | | | |
| | 4- تحسين مزاجي | | | | | |
| | **المقياس الفرعي : الصحة البدنية / اللياقة البدنية** | | | | | |
| | 5- تجعل من السهل القيام بالتمرين / الرياضة التي أستمتع بها | | | | | |
| | 6- تجعلني أشعر بمزيد من النشاط | | | | | |
| | 7- زيادة فرصتي في التمتع بصحة جيدة اآلن وفي المستقبل | | | | | |
| | 8- تحسين أعراض أو مشكلة صحية لدي اآلن | | | | | |
| | 9- تساعدني في أن أصبح أكثر لياقة بدنية ألتحسن في أداء وظيفتي | | | | | |
| | 10- تساعدني على النوم بشكل أفضل | | | | | |
| | 11- تجعل من السهل إنجاز أنشطتي اليومية | | | | | |

| | | لا أوافق مطلقا | لا أوافق | غير متأكد | أوافق | موافق تماما |
|---|---|---|---|---|---|---|
| | | | | | | |
| | المقياس الفرعي : الاجتماعي / المهني | | | | | |
| | 12- توفير فرصة أفضل للزواج. | | | | | |
| | 13- تجعلني أشعر براحة أكبر و انا حول الآخرين | | | | | |
| | **الدافع لفعل التغيير** | **لا أوافق مطلقا** | **لا أوافق** | **غير متأكد** | **أوافق** | **موافق تماما** |
| **سأتبنى عادات الأكل الصحي والنشاط البدني إذا ...** | **الدوافع الداخلية** | | | | | |
| | 1- نظرت في المرآة وكنت غير راضية عن جسدي | | | | | |
| | 2- ملابسي ضيقة بشكل غير مريح | | | | | |
| | 3- اصبت بمشكلة صحية يمكن تحسينها بوزن صحي. | | | | | |
| | عتقدت أن الآخرين يأخذون نظرة عني غير عادلة بنا [illegible] على وزني | | | | | |
| | 5- الوزن الصحي سيساعدني في تحقيق أهدافي الشخصية / المهنية | | | | | |
| | ن الصحي من شأنه أن يحسن من اكتئابي أو قلقي أو توتري | | | | | |
| **الدوافع الخارجية** | | | | | | |
| | 7- نصحني طبيب / ممرضة / اختصاصي تغذية أن أكون في وزن صحي | | | | | |
| | 8- أصيب أحد أفراد أسرتي بمشكلة صحية خطيرة بسبب زيادة الوزن أو السمنة | | | | | |

| | | | | | | |
|---|---|---|---|---|---|---|
| | 9- نصحني أحد أفراد األسرة أو صديق مقرب بأن أكون في وزن صحي | | | | | |
| | 10- قدمت لي معلومات عن الصحة مخاطر زيادة الوزن / السمنة في دورة 11- قرأت في الراديو أو التلفزيون أو وسائل التواصل االجتماعي موقع حول المخاطر الصحية لزيادة الوزن / السمنة | | | | | |
| | 12- رأيت إعالن [illegible]ا عن منتج أو خدمة ادعى أنها تساعدني في أن أكون في وزن صحي | | | | | |
| **الكفاءة الذاتية المتصورة في الرجيم** | | **لاوافق مطلقا** | **ال أوافق** | **غير متأكد** | **أوافق** | **موافق تما ما** |
| **المقياس الفرعي : العادات والتفضيالت المقياس الفرعي** | | | | | | |
| 1- يمكنني تناول ثالث وجبات بانتظام | | | | | | |
| 2- يمكنني تناول وجبات الطعام بكميات معتدلة. | | | | | | |
| 3- يمكنني أن آكل طعا[illegible]ما طاز جا بدال [illegible] من األغذية المصنعة | | | | | | |
| 4- يمكنني االمتناع عن تناول الحلويات مثل الحلوى والبسكويت. | | | | | | |
| 5- يمكنني االمتناع عن تناول األطعمة الدهنية مثل األطعمة المقلية. | | | | | | |
| 6- يمكنني االمتناع عن شرب المشروبات الغازية مثل الكوال | | | | | | |
| 7- يمكنني تناول أطعمة مختلفة لتجنب عدم التوازن في النظام الغذائي | | | | | | |
| 8- يمكنني االمتناع عن تناول الطعام قبل النوم مباشرة. | | | | | | |
| 9- أستطيع أن آكل ببطء حتى عندما أجوع | | | | | | |
| 10- يمكنني التوقف عن تناول الطعام قبل ملء المعدة حتى لو كان | | | | | | |

| | | | | | |
|---|---|---|---|---|---|
| الطعام لذيذ [illegible]ا. | | | | | |
| 11- يمكنني االستيقاظ مبكرا لتناول اإلفطار. | | | | | |
| 12- يمكنني االمتناع عن مشاهدة التلفزيون أو قراءة كتاب عندما آكل | | | | | |
| 13- يمكنني رفض تناول الطعام عندما يقدم لي أفراد عائلتي أو أصدقائي األطعمة. | | | | | |
| **المقياس الفرعي : الصحة العاطفية / العقلية** | | | | | |
| 14 أستطيع االمتناع عن األكل عندما أشعر بالملل. | | | | | |
| 15- يمكنني االمتناع عن تناول الطعام عندما أشعر بالجوع. | | | | | |
| 16- يمكنني االمتناع عن تناول الطعام عندما أكون غاضب [illegible]ا. | | | | | |
| 17- يمكنني االمتناع عن تناول الطعام عندما أشعر باالكتئاب. | | | | | |
| 18- يمكنني االمتناع عن تناول الطعام عندما أكون متوترة ومتحمسة. | | | | | |
| **الكفاءة الذاتية المتصورة في التمرين** | **لوافق مطلقا** | **ال أوافق** | **غير متأكد** | **أوافق** | **موافق تما ما** |
| 1- يمكنني المشي لمسافة تصل إلى 15 دقيقة سي [illegible]ا على األقدام. | | | | | |
| 2- يمكنني ممارسة الرياضة حتى أصاب بضيق في التنفس. | | | | | |
| 3- يمكنني ممارسة الرياضة حتى أصاب بضيق في التنفس. | | | | | |
| 4- يمكنني ممارسة الرياضة في الطقس الحار. | | | | | |
| 5- يمكنني ممارسة الرياضة مع األصدقاء بعد المدرسة. | | | | | |
| 6- يمكنني ممارسة الرياضة بدال [illegible] من مشاهدة التلفزيون في أوقات الفراغ | | | | | |
| 7- يمكنني استخدام الساللم بدال [illegible] من المصاعد. | | | | | |
| **النية السلوكية للتحكم في الوزن** | **لوافق مطلقا** | **ال أوافق** | **غير متأكد** | **أوافق** | **موافق تما ما** |
| **المقياس الفرعي : العالج الغذائي** | | | | | |
| 1- أعتزم التحكم في النظام الغذائي لتقليل الوزن في غضون ستة أشهر | | | | | |
| 2- أنوي زيارة اختصاصي تغذية لتقليل الوزن في غضون ستة أشهر. | | | | | |
| 3- أنوي حضور فصل السمنة إذا فتحته مدرستي. | | | | | |
| **المقياس الفرعي : العالج بالتمرين** | | | | | |

| | | | | | |
|---|---|---|---|---|---|
| 4- أنوي ممارسة الرياضة بنفسي بانتظام لتقليل الوزن ستة أشهر. | | | | | |
| 5- أنوي حضور مركز أو فصل رياضي لتقليل الوزن في غضون ستة الشهور | | | | | |

شكرا لتعاونك في الإجابة على هذا الاستبيان.

**SCHOOLS NAMES, ADDRESS AND TELEPHONE NUMBOR**

1. **SCHOOL 1: ELITE ARABIC SCHOOL**

   ADDRESS: Jalan Tenaga, Taman Tenaga,43000 Kajang, Selangor

   TELE NO. :0172677665

2. **SCHOOL 2: Saudi Schools In Kuala Lumpur**

   ADDRESS: Jalan Hulu Kelang, Taman Zooview, 68000 Ampang, Selangor

   TELE NO. :0341055531

3. **SCHOOL 3: Global Modern International School**

   ADDRESS: No.1, Jalan Sinar Pagi, Country Heights, 43000 Kajang, Selangor

   TELE NO. : 03-8730 1444

4. **SCHOOL 4: International Modern Arabic School**

   ADDRESS: Jalan P14k, Presint 14, 62050 Putrajaya, Wilayah Persekutuan Putrajaya

   TELE NO. : 03-8888 5388
