# Supplementary material for: Effectiveness of education intervention, with regards to physical activity level and a healthy diet, among Middle Eastern adolescents in Malaysia: A study protocol for a randomized control trial, based on a health belief model
Source: PLoS One. 2024 Jan 17;19(1):e0289937. doi: 10.1371/journal.pone.0289937 (PMC10793934; doi:10.1371/journal.pone.0289937)
Supplement: Attachment — Submitted filename: Response to Reviewers.docx [file pone.0289937.s003.docx]

**RESPONSE TO REVIEWERS**

**Effectiveness of education intervention, with regards to physical activity level and a healthy diet, among Middle Eastern adolescents in Malaysia: a study protocol for a randomized control trial, based on a health belief model**

Dear editors,

We thank all reviewers for their valuable comments. We have modified the manuscript according to each of the respective reviewers. We hope that the reviewers and editors will satisfy with the amendments in the revised manuscript. Details are listed below:

| **Reviewer’s comments** | **Author responses** | **Pages and Lines** |
| --- | --- | --- |
| **Editor's Comments** | | |
| 1. You need to extended more details about CONSORT. See: Consort 2010 statement: extension to cluster randomised trials. | We add more details about the CONSORT. | **Page 8**  **Lines 238-240** |
| 2. Do the authors have access to further baseline data such as, e.g highest education level for parents, or ethnicity, as this might also play a role in confounding with outcome. | We add the ethnicity, highest education level for parents, parents' occupation. | **Page 17**  **Lines 446-448** |
| 3. As the program was 6 weeks, was compliance measured in any way? | The compliance will not be measured. However, participants only will receive motivational messages. | **Page 14**  **Lines 301-304** |
| 4. There are more similar studies therefore this is not the first intervention study. So the claim is false and should be changed after review. | We removed the sentence. | **Page 21**  **Lines 458-467** |
| 5. It is good that HBM model is being used. But there seems that only one model has been reviewed for developing intervention. The suggestion is that to read more models, look for advantages and disadvantages and then select. Even a multi-model strategy has been used for developing intervention. For example, WHO has suggested one TMT, BCW is another one. You need to review all the models and choose the model/s that are more appropriate in your context. | We include more information regarding the model with the advantages and disadvantages. | **Pages 6-7**  **Lines 123-158** |
| 6. Must read about issues and problems of using HBM before rejecting it or using it along with other models. | We include more information regarding the model with the advantages and disadvantages. | **Page 6**  **Lines 129-136** |
| **Reviewer 1** | | |
| The authors have mentioned following the CONSORT. Can this be extended to look at the Research Methods & Reporting, Consort 2010 statement: extension to cluster randomised trials. | We add more details about the CONSORT. | **Page 8**  **Lines 238-240** |
| In addition do the authors have access to further baseline data such as, e.g highest education level for parents, or ethnicity, as this might also play a role in confounding with outcome. | We add the ethnicity, highest education level for parents, parents' occupation. | **Page 17**  **Lines 446-448** |
| As the program was 6 weeks, was compliance measured in any way? | The compliance will not be measured. However, participants only will receive motivational messages. | **Page 14**  **Lines 301-304** |
| **Reviewer 2** | | |
| 1. There are more similar studies therefore this is not the first intervention study. So the claim is false and should be changed after review. | We removed the sentence. | **Page 21**  **Lines 458-467** |
| 2. It is good that HBM model is being used. But there seems that only one model has been reviewed for developing intervention. I would suggest that read more models, look for advantages and disadvantages and then select. Even a multi-model strategy has been used for developing intervention. For example, WHO has suggested one TMT, BCW is another one, but I would encourage you to review all the models and choose the model/s that are more appropriate in your context. | We include more information regarding the model with the advantages and disadvantages. | **Pages 6-7**  **Lines 123-158** |
| Must read about issues and problems of using HBM before rejecting it or using it along with other models. | We include more information regarding the model with the advantages and disadvantages. | **Page 6**  **Lines 129-136** |
