# Supplementary material for: Effectiveness of education intervention, with regards to physical activity level and a healthy diet, among Middle Eastern adolescents in Malaysia: A study protocol for a randomized control trial, based on a health belief model
Source: PLoS One. 2024 Jan 17;19(1):e0289937. doi: 10.1371/journal.pone.0289937 (PMC10793934; doi:10.1371/journal.pone.0289937)
Supplement: Attachment — Submitted filename: Response to Reviewers.docx [file pone.0289937.s004.docx]

**RESPONSE TO REVIEWERS**

**Effectiveness of education intervention, with regards to physical activity level and a healthy diet, among Middle Eastern adolescents in Malaysia: a study protocol for a randomized control trial, based on a health belief model**

Dear editors,

We thank all reviewers for their valuable comments. We have modified the manuscript according to each of the respective reviewers. We hope that the reviewers and editors will satisfy with the amendments in the revised manuscript. Details are listed below:

| **Reviewer’s comments** | **Author responses** | **Pages and Lines** |
| --- | --- | --- |
| **Editor's Comments** | | |
| 1. The CONSORT why you choose this guideline not others.?. | The CONSORT 2010 guideline were chosen because it is intended to improve the reporting of parallel- group randomized controlled trials. It enables better understanding of a trial design, conduct, analysis, interpretation and assessment of the validity of its results. | **Page 10**  **Lines 238-240** |
| 2. You need to answer the question: Do the authors have access to further baseline data such as, e.g highest education level for parents, or ethnicity, as this might also play a role in confounding with outcome? By saying whether the authors have access or not and what are these data. | The authors have access to further baseline data including ethnicity, highest education level for parents, parents' occupation. | **Page 20**  **Lines 449-451** |
| 1. Please ensure that you refer to Figure 2 in your text as, if accepted, production will need this reference to link the reader to the figure. | Corrected | **Page 11**  **Lines 238** |
| 2. Figure 3 is cited but not provided. If the figure is no longer to be included as part of the submission please remove all reference to it within the text. | Corrected No figure 3 |  |
